# APOL1 polymorphism modulates sphingolipid profile of human podocytes

**DOI:** 10.1007/s10719-020-09944-w

**Published:** 2020-09-11

**Authors:** Manuela Valsecchi, Valentina Cazzetta, Ferdinando Oriolo, Xiqian Lan, Rocco Piazza, Moin A. Saleem, Pravin C. Singhal, Domenico Mavilio, Joanna Mikulak, Massimo Aureli

**Affiliations:** 1 Department of Medical Biotechnologies and Translational Medicine (BioMeTra), University of Milan, Milan, Italy; 2 Unit of Clinical and Experimental Immunology, Humanitas Clinical and Research Center – IRCCS, Rozzano, MI Italy; 3 Key Laboratory for Aging and Regenerative Medicine, School of Pharmacy, Southwest Medical University, Luzhou, Sichuan China; 4 Department of Medicine and Surgery, University of Milan-Bicocca, Monza, Italy; 5 Pediatric Academic Renal Unit, University of Bristol, Bristol, UK; 6 Institute of Molecular Medicine, Feinstein Institute for Medical Research and Zucker School of Medicine at Hofstra-Northwell, Hempstead, NY USA

**Keywords:** APOL1, *APOL1* polymorphism, HIVAN, Sphingolipids, Podocytes, Lipid rafts, Plasma membrane

## Abstract

Apolipoprotein L1 (APOL1) wild type (G0) plays a role in the metabolism of sphingolipids, glycosphingolipids, sphingomyelin and ceramide, which constitute bioactive components of the lipid rafts (DRM). We asked whether APOL1 variants (APOL1-Vs) G1 and G2 carry the potential to alter the metabolism of sphingolipids in human podocytes. The sphingolipid pattern in HPs overexpressing either APOL1G0 or APOL1-Vs was analysed by using a thin mono- and bi-dimensional layer chromatography, mass-spectrometry and metabolic labelling with [1-^3^H]sphingosine. HP G0 and G1/G2-Vs exhibit a comparable decrease in lactosylceramide and an increase in the globotriaosylceramide content. An analysis of the main glycohydrolases activity involved in glycosphingolipid catabolism showed an overall decrease in the activeness of the tested enzymes, irrespective of the type of APOL1-Vs expression. Similarly, the high throughput cell live-based assay showed a comparable increased action of the plasma membrane glycosphingolipid-glycohydrolases in living cells independent of the genetic APOL1 expression profile. Importantly, the most significative modification of the sphingolipid pattern induced by APOL1-Vs occurred in DRM resulted with a drastic reduction of radioactivity associated with sphingolipids. G1/G2-Vs present a decrease amount of globotriaosylceramide and globopentaosylceramide compared to G0. Additionally, ceramide at the DRM site and lactosylceramide in general, showed a greatest fall in G1/G2 in comparison with G0. Additionally, the levels of glucosylceramide decreased only in the DRM of human podocytes overexpressing G1/G2-Vs. These findings suggest that altered sphingolipidsprofiles may contribute to the deranged functionality of the plasma membrane in APOL1 risk milieu.

**Electronic supplementary material:**

The online version of this article (10.1007/s10719-020-09944-w) contains supplementary material, which is available to authorized users.

## Introduction

Apolipoprotein L1 (APOL1) is a minor component of plasma circulating High-Density Lipoprotein (HDL) capable to kill *Trypanosoma brucei* responsible for African sleeping sickness. [1–5]. The emerging resistance of the two specific variants (Vs) of APOL1 gene, termed G1 (rs73885319, p.S342G) and G2 (rs71785313, p.N388_Y389del), to the Trypanosoma brucei gambiense and rhodesiense infection increased their frequency in the residents of many regions of Africa as a consequence of pathogen positive selection [6]. Nevertheless, these two *G1* and *G2 APOL1 gene* Vs, in contrast to the wild type (WT) *G0* allele, have been shown to associate with several kidney pathologies including hypertension-attributed nephropathy, non-diabetic end-stage kidney disease, and the most strongly focal segmental glomerulosclerosis (FSGS) and HIV-associated nephropathy (HIVAN), an important complication of HIV infection characterized by collapsing FSGS associated to massive proteinuria [7, 8]. The pathogenesis of HIVAN is likely due to direct HIV infection of podocytes, the end-stage differentiated kidney epithelial cells critical for the glomerular filtration barrier [9, 10]. Moreover, capturing of HIV by human podocytes (HPs) contribute to the establishment of the renal HIV reservoir important for viral spreading through trans-infection to lymphocytes [11].

APOL1 is a lipid-binding protein constitutively express in HPs and relevant for cellular homeostasis through endosomal trafficking and autophagy regulation, and activation of the inflammatory and innate immune response [12–15]. Many studies have already established that APOL1 risk Vs induce podocytes injury through mechanisms including increase of the lysosomal membrane permeability, mitochondrial dysfunction and impairment of endosomal and autophagic trafficking [16–20]. Additionally, we have previously shown that intracellular levels of HIV accumulation in HPs relies on expression of the APOL1 G1/G2-Vs upon inflammatory conditions [21]. However, there may be additional mechanisms by which APOL1-Vs injure HPs.

Taken together, these considerations let to speculate that plasma membrane could play an important role. In particular, among membrane lipids, sphingolipids (SLs) are directly involved in the regulation of the membrane homeostasis and signalling by the organization of specific macromolecular complex called lipid rafts. SLs are critical for proper function of the glomerular filtration barrier by regulating several podocytes functions such as: i) cell-to-cell interactions [22–26]; ii) cell survival and proliferation [26–28]; iii) endosomal trafficking [28, 29], and iv) pathogen capture including HIV [30, 31]. The family of the SLs include different classes of lipids with a varying degree of hydrophobic and hydrophilic proprieties. The hydrophobic region of SLs consists of a long chain sphingoid base, which is linked to a fatty acid via an amide bond. On the other hand, the hydrophilic portion could be composed by a hydroxyl group such as in the case of ceramide (Cer), phosphocholine in the case of sphingomyelin (SM) or by the combination of different saccharides forming the group of glycosphingolipids (GSLs). The diversity of GSLs is directed by a range of proteins involved in glycan biosynthesis including glycosyltransferases (GTs) and glycosidases, enzymes involved in glycan precursor biosynthesis and sugar transporters. These enzymes are diversely expressed in different types of human cells in order to achieve the cell specific SL plasma membrane functions. Indeed, alterations in the SLs pattern are usually associated with pathological conditions including podocyte injury [32].

**Fig. 1:**
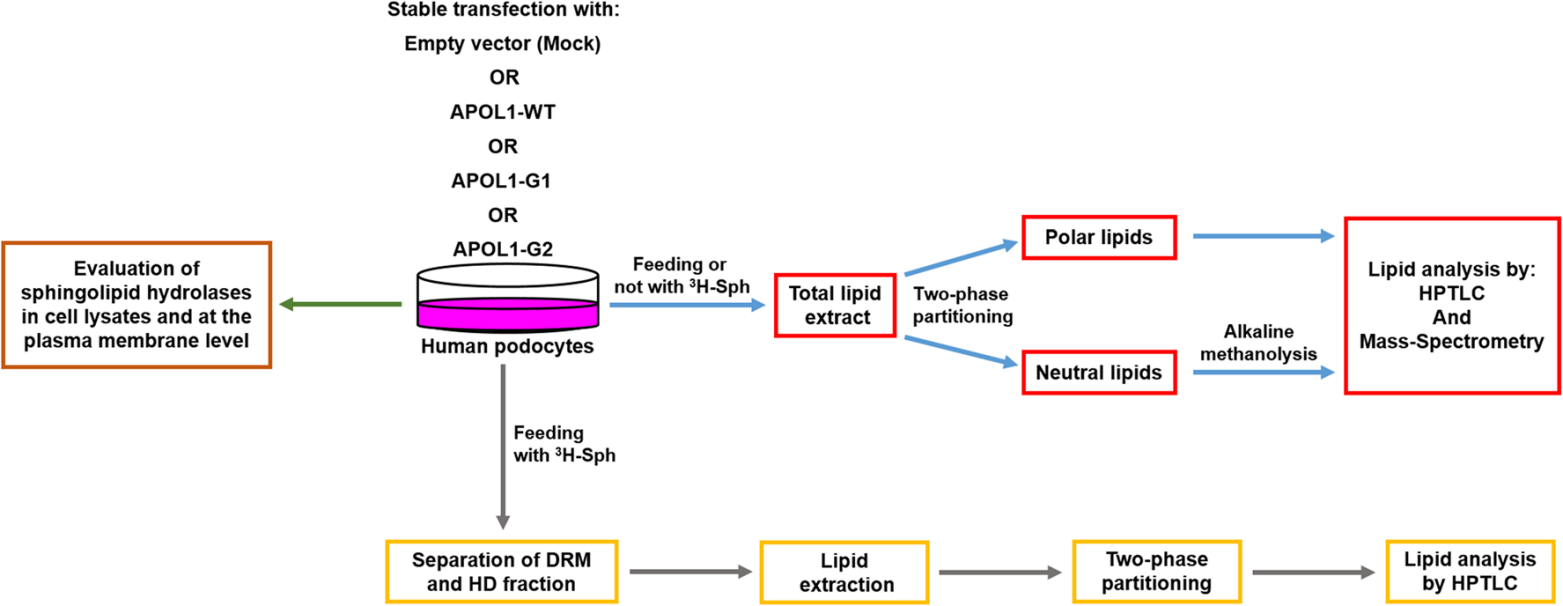
Experimental pipeline followed to characterize podocytes expressing the different APOL1 Vs in term of SL composition and of the hydrolases involved in their catabolism

Nevertheless, the genetic associations between APOL1-Vs and SL metabolism in HPs have not been investigated yet. Here we propose a study, schematically summarized in fig. 1, aimed to characterize the effect of the expression of APOL1 and its pathological variants on HPs in term of SL pattern and their catabolic enzymes.

## Results

### Sphingolipid profile of human podocytes

One of the main challenges in studying primary HPs is due to their terminally differentiated phenotype that has been overcame in part, by the *in vitro* development of conditionally immortalized HPs. This approach has greatly advanced our understanding of the physiopathology of HPs [33–35]; therefore, we took advantage of this established *in vitro* model to examine their SL profile. The analysis of SL pattern has been evaluated by combining mono- and bi-dimensional thin layer chromatography (HPTLC), mass-spectrometry (MS) and the metabolic tritium labelling at the steady state with [1-^3^H]sphingosine ([1-^3^H]Sph). As expected, the SL pattern of HPs identified by HPTLC and MS analysis covers both neutral and polar SL species. Among analyzed complex SLs, the most abundant chain lengths of fatty acids present in HPs and their unsaturation degree were C16:0, C22:0, C24:1 and C24:0. The list and structure of all identified SLs in HPs is reported in supplementary Table 1.

The endogenous and the metabolically tritium labeled SL pattern is reported in supplementary Fig. 1. Seven neutral SLs, covering over 85% of the total SLs, namely, ceramide, glucosylceramide, lactosylceramide, globotrihexosylceramide, ganglioteraosylceramide, sphingomyelin and globopentaosylceramide were identified by MS and comparison with authentic standards. Sphingomyelin is the major neutral SL in HPs, constituting over 26% of total SL content. Among the acidic SLs, the most representative is the ganglioside GM_3_, covering near 5% of total SLs. Moreover, we identified GM_1b_ and GD_1α_, and the mono and disialylated forms of globopentaosylceramide (Gb_5_Cer). Quantification of the single SL types carried out on both endogenous and metabolically tritium labeled SLs resulted in comparable results. Indeed, as is shown in supplementary Fig. 1, a similar pattern and distribution of SLs were observed in both endogenous and metabolically tritium labeled SLs.

Common to other human cell types, also in HPs the main SL among non polar compounds are sphingomyelin (26.3% ± 2.8% of total cell SLs) and GM_3_ between ganglioside species (5.2% ± 0.3%). In total SLs we also found globopentaosylceramide (4.9% ± 0.9%) and its two sialylated forms, monosialosyl-globopentaosylceramide and disialosyl-globopentaosylceramide, that represent 4.5% ± 0.3% and 0.4% ± 0.2%, respectively. These structures do not belong to the isogloboseries due to the lack of expression in HPs of *A3GALT2* gene encoding for this enzyme. In fact, gene expression level of *A3GALT2* was undetectable in whole-transcriptome analysis generated in human immortalized podocytes (HPs) and downloaded from the NCBI SRA database (*accession number: SUB7456861*; data not shown). On the other hand, a high expression level of *A4GALT* gene was revealed*,* thus indicating that detected globopentaosylceramides in HPs belong to the globoseries (data not shown). The GA1 reaches 2.4% ± 0.4% of all SLs, and the gangliosides GM_1b_ and GD_1α_, constitute respectively 3.4% ± 0.4% and 1% ± 0.3% among all SLs. In addition, whole transcriptional data analysis in HPs evidenced gene expression of *B4GALNT1* enzyme, thus confirming the presence of 0-series gangliosides. The distribution of neutral SLs in HPs represents the following percentages listed in raising order: ceramide 5.7% ± 1%; glucosylceramide 12.5% ± 2.1%; lactosylceramide 15.6% ± 1.7%, and globotriaosylceramide 18% ± 2.6%.

The mass spectra (MS1) with its corresponding fingerprints (MS2) shown in supplementary Fig. 2, confirmed the presence of 0- and α-series gangliosides in these cells and allowed to discriminate between the ganglio and globo series.

In supplementary Fig. 2, the sequence of ions at *m*/*z* 1626.90, 1335.79, 1173.75, 970.67, 808.62 and 646.56, suggests a Neu5Ac-Hex-HexNHAc-Hex-Hex-Cer structure characteristic of GM_1,_ with the lack of the fragment ion at *m*/*z* 1261.78 that is characteristic for the GM_3_ structure Neu5Ac-Hex-Hex-Cer. Moreover, when this ganglioside was treated with *V. Cholera* sialidase, GM_1_ converted to gangliotetraosylceramide (data not shown). The *V. Cholera* sialidase *in vitro* does not act on the inner sialic acid, confirming the position of the residue of sialic acid, bound to the terminal Gal. Taken all together, these results indicate that the sialidase-labile GM_1_ is GM_1b_, as also suggested by others [25, 36]. Similarly, GD_1_ represented by the MS2 spectrum derived from the ion at *m*/*z* 945.51, coincident with its doubly charged ion, contains the ions corresponding to Neu5Ac at *m*/*z* 290.11, (Neu5Ac-)HexNHAc at *m*/*z* 493.19, (Neu5Ac-)Hex-HexNHAc-Hex at *m*/*z* 835.29 and (Neu5Ac-)Hex-(Neu5Ac-)HexNHAc at *m*/*z* 964.35 respectively. As already reported [36], we didn’t detect the fragment ion corresponding to GM_3_ at *m*/*z* 1235.81, present in the case of GD_1a_, and no fragment ion corresponding to (Neu5Ac-)_2_ at *m*/*z* 581.22 was found, a typical fragment ion of GD_1b_. Moreover, upon the treatment with *V. Cholera* sialidase it was converted to GM_1_ and gangliotetraosylceramide (data not shown), confirming that sialic acids were bound to the terminal Gal and to the adjacent GalNAc, respectively. All these data indicate that the sialidase-labile GD_1_ is GD_1α_.

Regarding the globo-series of SLs (Gb_5_Cer, Neu5AcGb_5_Cer and (Neu5Ac)_2_Gb_5_Cer), their identification was based on the typical sequential fragmentation of their oligosaccharide chain. For Gb_5_Cer, the MS2 spectrum derived from the ion at *m*/*z* 1387.92 showed a series of ions at *m*/*z* 1225.87, 1022.79, 860.74, 698.68 and 536.63, corresponding to the sequential detachment of sugar moieties. Furthermore, the MS2 spectrum derived from the ion at *m*/*z* 895.00, corresponding to the doubly charged ion of Neu5AcGb_5_Cer, contained the ions corresponding to Neu5Ac at *m*/*z* 290.11, (Neu5Ac-)Hex at *m*/*z* 470.16 and (Neu5Ac-)Hex-HexNHAc-Hex at *m*/*z* 835.29, respectively, as previously described [37]. For (Neu5Ac)_2_Gb_5_Cer, in the MS2 spectrum derived from the ion at *m*/*z* 1039.56, corresponding to the doubly charged ion of (Neu5Ac)_2_Gb_5_Cer, the presence of the ions corresponding to Neu5Ac at *m*/*z* 290.11, (Neu5Ac-)HexNHAc at *m*/*z* 493.19 and (Neu5Ac-)HexNHAc-Hex-Hex at *m*/*z* 835.29, respectively, is consistent with its structure already reported in literature [38, 39]. When sialylated forms of Gb_5_Cer were treated with *V. Cholera* sialidase, they converted to GA1 (data not shown), confirming that sialic acids are linked to the terminal Gal in MSGb_5_Cer and in DSGb_5_Cer, and to the adjacent GalNAc in (Neu5Ac)_2_Gb_5_Cer. To further confirm the structure of (Neu5Ac)_2_Gb_5_Cer we did not find the fragment ion (Neu5Ac-)_2_ at *m*/*z* 581.22. Of note, we did not detect GD_3_ or O-acetylated-GD_3_ previously described within the rat podocytes, probably because of the specie-specific differences in the expression of some gangliosides between rodents and humans [40, 41].

### The overexpression in human podocytes of WT and G1 and G2 APOL1-Vs has differential effects on sphingolipids pattern and metabolism

**Fig. 2:**
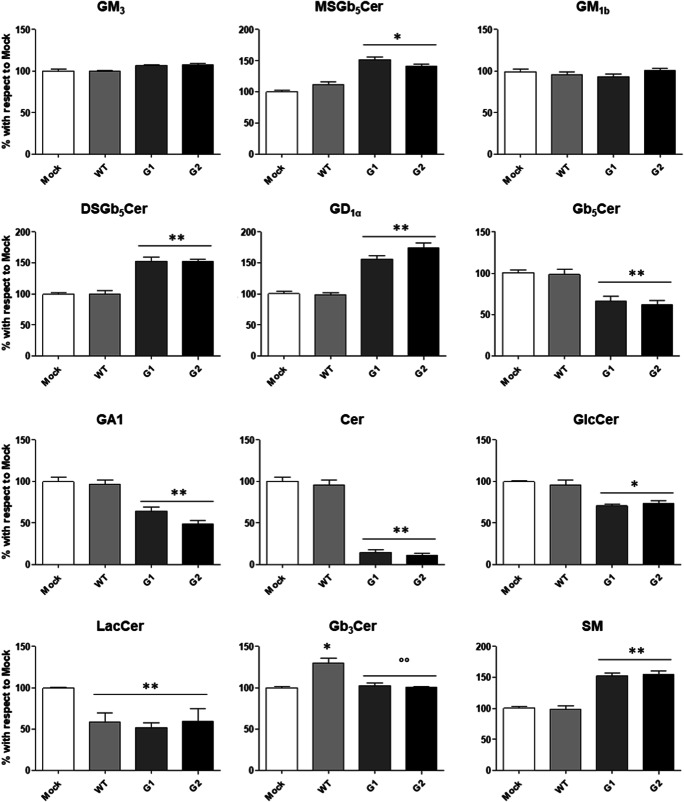
**Effects of the overexpression of APOL1 WT, G1 and G2 Vs on the sphingolipid pattern of human podocytes.** Cell sphingolipids were metabolically labelled at the steady state with [1-^3^H]sphingosine, separated by HPTLC, visualized by digital autoradiography and quantified with M3 software. Data are expressed as percentage with respect to Mock cells. Gangliotetraosylceramide (GA1), globotriaosylceramide (Gb_3_Cer), globopentaosylceramide (Gb_5_Cer), monosialosyl-globopentaosylceramide (MSGb_5_Cer), disialosyl-globopentaosylceramide (DSGb_5_Cer), ceramide (Cer), glucosylceramide (GlcCer), lactosylceramide (LacCer), sphingomyelin (SM). *p < 0.05 vs Mock, **p < 0.005 vs Mock, °° p < 0.005 vs WT

APOL1 is an integral component of HDL particles that might be involved in cholesterol efflux from the cell, membrane homeostasis, oxidative stress, phospholipid transport and regulation of intracellular processes, including autophagy and vesicle transport. However, studies to shed light on the role of WT and the specific APOL1-Vs on SLs homeostasis in HPs under both physiological and pathological conditions are needed. Therefore, we use the HPs *in vitro* model overexpressing WT APOL1 or either the two G1/G2 APOL1-Vs to study the metabolism of SLs. We have validated an equivalent *APOL1* gene expression level in all HP samples by real time PCR analysis (data not shown) and by whole-transcriptome data generated in these cells (*NCBI SRA database; accession number: SUB7456861*) and reported in supplementary Fig. 3. By the analyses of the SL profile, we observed that the stable overexpression of the WT form of APOL1 in HPs induce a decrease in the total cell content of the lactosylceramide of about 42% ± 6% and an increase of the 30% ± 3% of the globotriaosylceramide (Fig. 2). Interestingly, the overexpression of the risk proteins of APOL1 G1 and G2 induce similar changes in almost all the podocyte SLs with the exception for the gangliosides GM_3_, GM_1b_ and globotriaosylceramide. Indeed, the overexpression of G1 and G2 Vs induces an increase of: 50% ± 3% for the monosialosyl-globopentaosylceramide, 53% ± 2% for the disialosyl-globopentaosylceramide, 58% ± 9% for the ganglioside GD_1α_, and 52% ± 3% for the sphingomyelin. On the contrary, the overexpression of the two APOL1-Vs induces a decrease of: 34% ± 4% for the globopentaosylceramide, 36% ± 2% for the GA1, 86% ± 19% for the ceramide, 29% ± 2% for the glucosylceramide, and 42% ± 6% for the lactosylceramide.

**Fig. 3:**
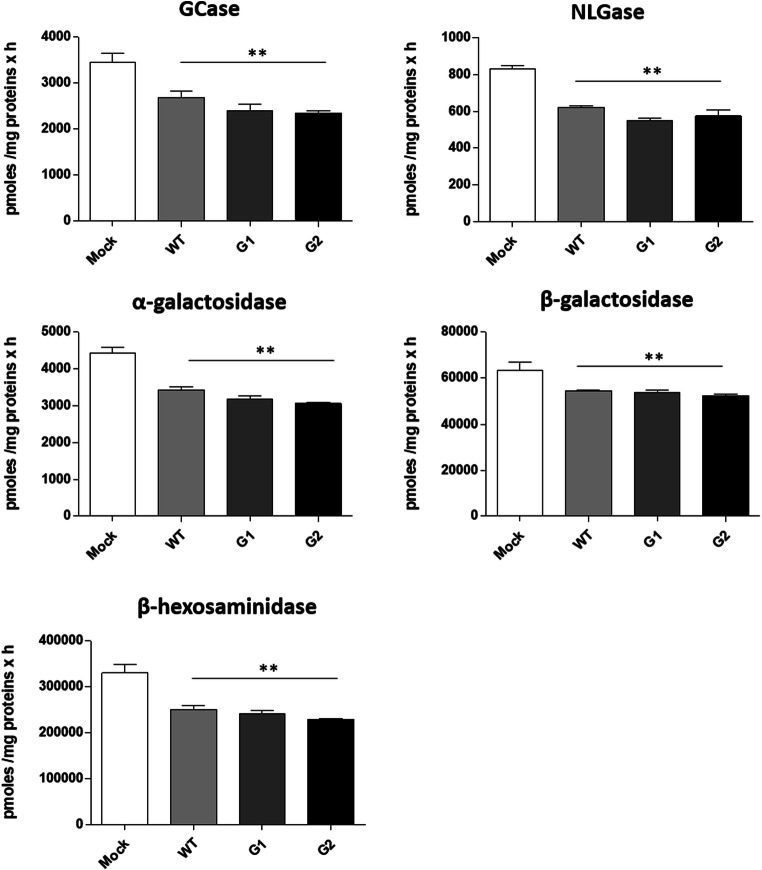
**Effects of the overexpression of APOL1 WT, G1 and G2 Vs on the activity of total cell sphingolipid-hydrolases.** Hydrolases activity were evaluated on cell lysate by an *in vitro* enzymatic assay using artificial fluorogenic substrates. β-glucocerebrosidase (Gcase), non-lysosomal glucosylceramidase (NLGase). The enzymatic activities are expressed as pmoles /mg of cell proteins per hour. **p < 0.005 vs Mock

We than analysed the activity of the main glycohydrolases involved in the glycosphingolipid catabolism. As is shown in Fig. 3, a significant decrease in the activity of the tested enzymes, independently on the overexpression of WT or APOL1-Vs, were found.

**Fig. 4:**
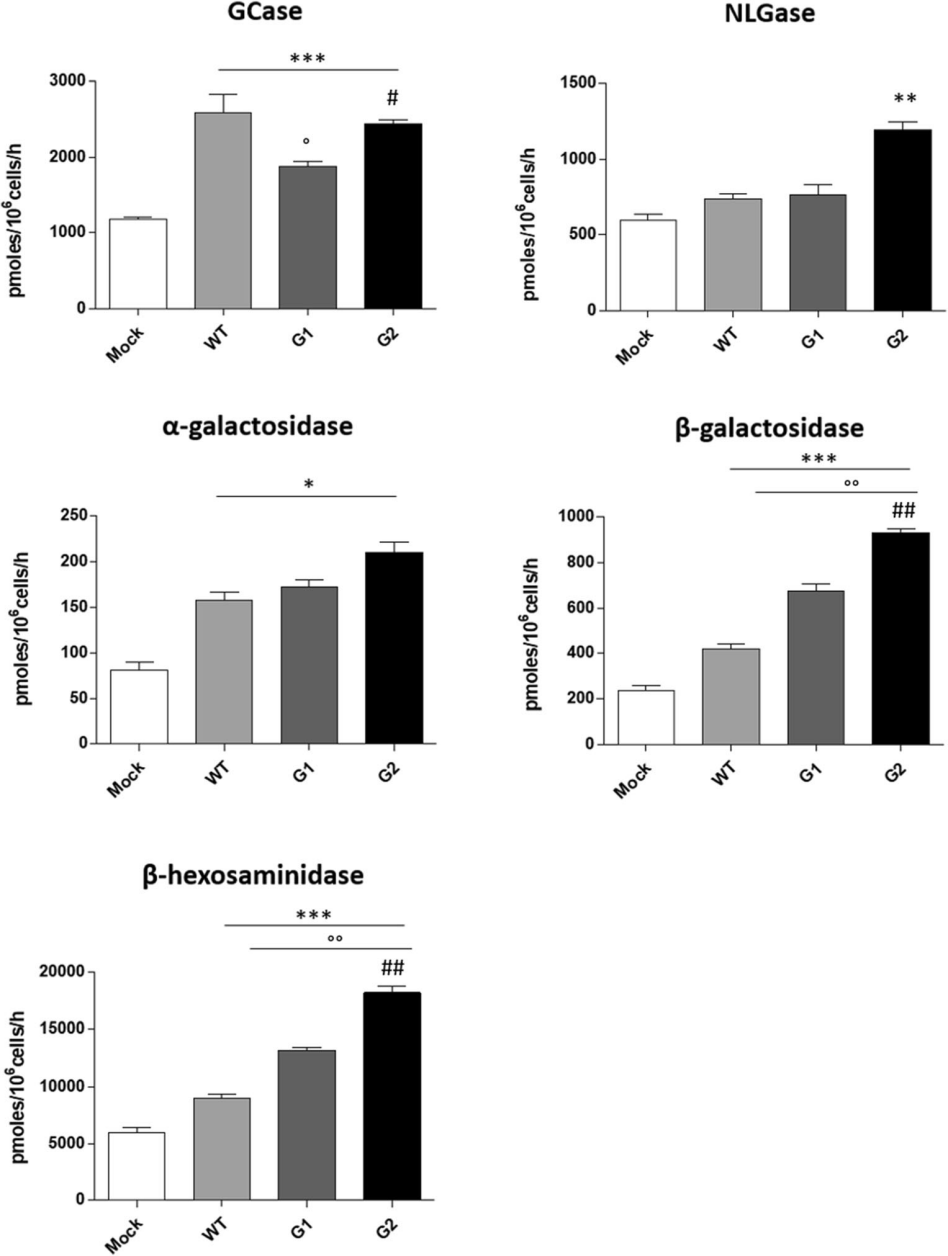
**Effects of the overexpression of APOL1 WT, G1 and G2 Vs on the activity of the sphingolipid-hydrolases associated with plasma membrane.** Hydrolases activity were evaluated at the plasma membrane on living cells using artificial fluorogenic substrates. β-glucocerebrosidase (Gcase), non-lysosomal glucosylceramidase (NLGase). The enzymatic activities are expressed as pmoles /10^6^ cell per hour. *p < 0.05, vs Mock **p < 0.005 vs Mock, *** *p* < 0.0005 vs Mock, ° p < 0.05 vs WT, °° p < 0.005 vs WT, # p < 0.005 vs G1, ## p < 0.005 vs G1

The same glycohydrolases enzymes are also located at the cell surface, where they are directly involved in the *in situ* modification of the SL composition at the plasma membrane. By a high throughput cell live-based assay, we measured the enzymatic activity of the sphingolipid-glycohydrolases at the plasma membrane site directly on living cells. Interestingly, as shown in Fig. 4 we found an increased activity for all the enzymes tested. Notable, the non-lysosomal beta-glucosylceramidase (NLGase) increased, only in case of the specific G2 risk variant overexpression. These data strongly suggest that the changes observed in the lipid composition after overexpression of the WT and APOL1-Vs could also be due to the action of the glycosphingolipid-hydrolases located at the cell membrane surface.

### The overexpression of APOL1 WT, G1 and G2 Vs alters the lipid rafts composition of human podocytes

**Fig. 5:**
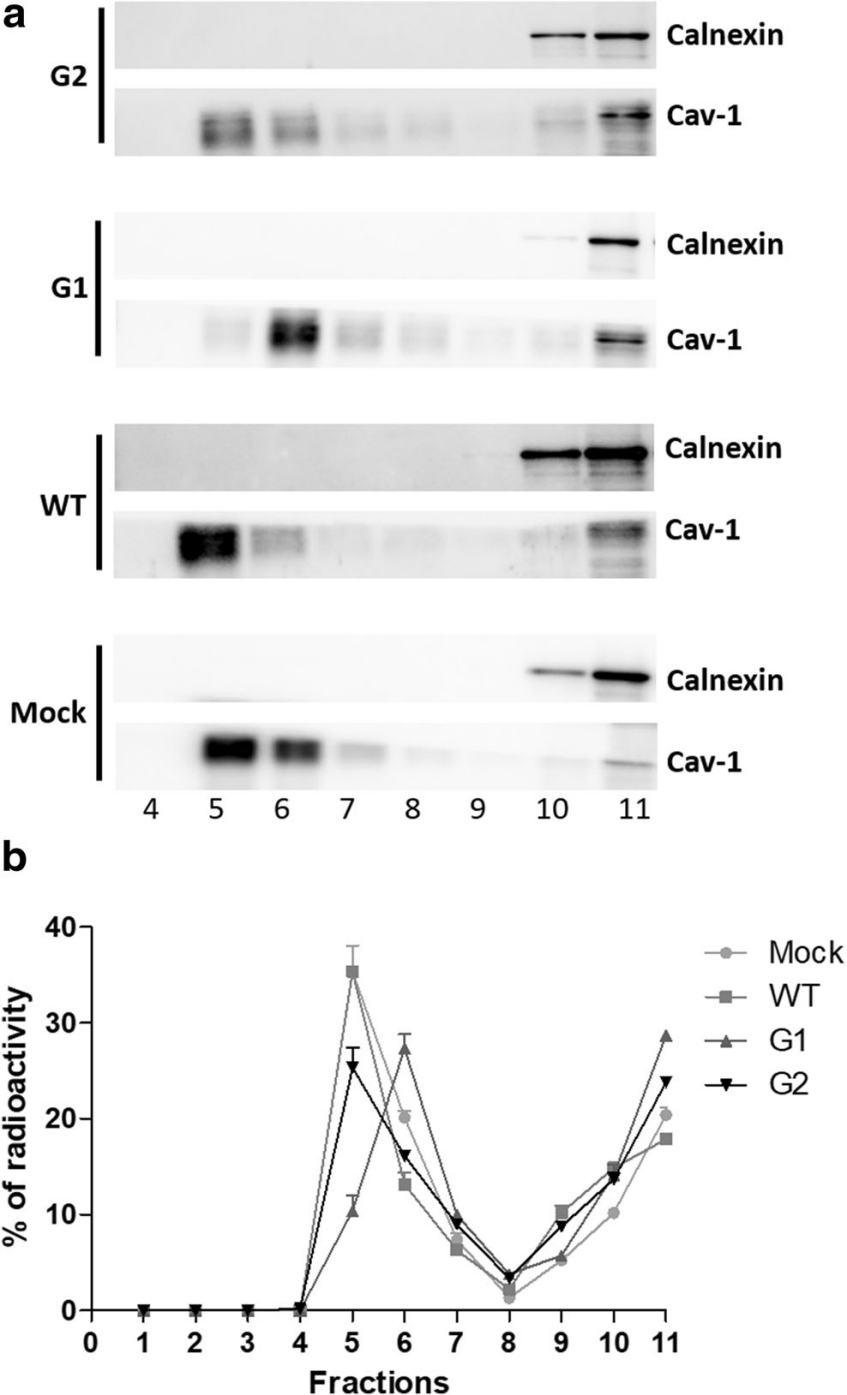
**DRM separation from podocytes overexpressing APOL1 WT, G1 and G2 Vs.** Cell sphingolipids were metabolically labelled at the steady state with [1-^3^H]sphingosine and DRM were separated on discontinuous sucrose gradient loading the same amount of cell lysates and corresponding to 2 mg of cell proteins and 225 nCi of radioactivity. A) Representative western blot of calnexin (not DRM marker) and caveolin-1 (Cav-1, DRM marker) in the gradient fractions obtained by the different cell line analyzed. B) Distribution of radioactivity among gradient fractions

The podocyte slit diaphragm, which has a critical role in the formation and maintenance of the glomerular filtration barrier, is assembled in the so called lipids rafts or detergent resistant membrane fractions (DRM). These small (10–200 nm diameter) specialized plasma membrane domains are enriched with SLs, cholesterol and protein complexes that have different roles in signal transduction and cell homeostasis. In the context of HIV infection, our previous work has shown that cholesterol (maintenance of DRM integrity) is required for HIV internalization in HPs [30]. In addition, as described before, we found that the overexpression of the APOL1-Vs strongly impacts the SL pattern of the HPs. Based on these considerations, mock HPs and overexpressing WT or G1/G2-Vs of APOL1 were fed with radioactive sphingosine in order to obtain the metabolic labelling at the steady state of all cell SLs. Subsequently DRM were isolated from plasma membrane and other cellular fractions on a discontinuous sucrose gradient as described in the methods. Eleven fractions were collected and analysed by Western blot for the positive (caveolin-1) and negative (calnexin) DRM markers. As shown in Fig. 5A, we found that fraction 5 and 6 are enriched in the DRM marker Caveolin-1 (Cav-1) compared to the high-density fractions 10 and 11. On the contrary, calnexin, a protein not associated with DRM, was undetectable in fractions 5 and 6 while it was detected in fractions 10 and 11. We than evaluated the radioactivity associated with each faction as an indicator of the SL distribution (Fig. 5B). We observed that mock and podocytes overexpressing WT APOL1 are characterized by similar radioactivity associated with SLs in the DRM fractions 5 and 6, whereas in DRM, isolated from HPs overexpressing the risk APOL1-Vs, the SL associated-radioactivity was reduced, thus suggesting that the overexpression of the pathological APOL1-Vs might negatively influence the SL composition of DRM in HPs.

**Fig. 6:**
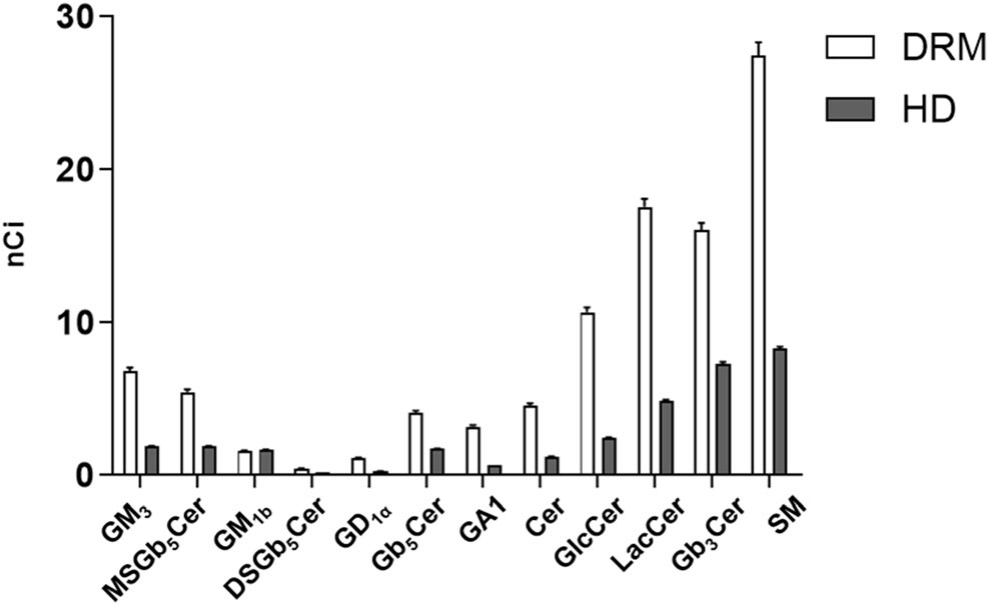
**Sphingolipid distribution among lipids rafts and detergent-soluble membrane fractions obtained from human podocytes.** Cell sphingolipids were metabolically labelled at the steady state with [1-^3^H]sphingosine and lipid rafts (DRM) and high-density fraction (HD) were separated after cell lysis with 1% TX 100 at 4 °C followed by ultracentrifugation on a discontinuous sucrose gradient. Radioactive lipids were extracted, separated with HPTLC, visualized by digital autoradiography and quantified with M3 software. In the graphs, the data were reported as nCi associated to each lipid. Gangliotetraosylceramide (GA1), globotriaosylceramide (Gb_3_Cer), globopentaosylceramide (Gb_5_Cer), monosialo-globopentaosylceramide (MSGb_5_Cer), disialo-globopentaosylceramide (DSGb_5_Cer), ceramide (Cer), glucosylceramide (GlcCer), lactosylceramide (LacCer), sphingomyelin (SM). *p* < 0.0005

Further analysis of the SL pattern associated specifically with the DRM was performed on the fractions 5 and 6 pulled together and compared with the pulled fractions 10 and 11 of the detergent soluble membrane. The DRM obtained from mock HPs are enriched in the amount of almost all SLs, with exception of the GM_1b_ which is present at the same level observed in the detergent soluble fractions (Fig. 6).

**Fig. 7:**
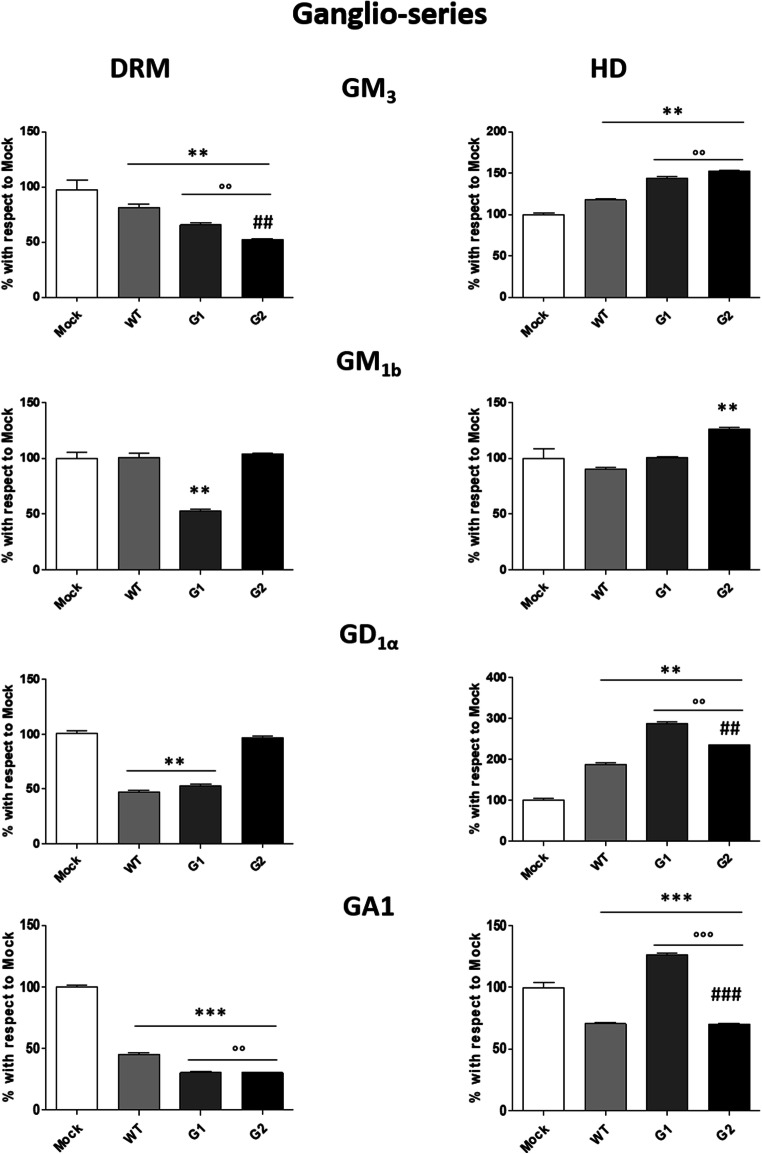
**Effects of the overexpression of APOL1 WT, G1 and G2 Vs on the ganglio-series content of lipid rafts.** Cell sphingolipids were metabolically labelled at the steady state with [1-^3^H]sphingosine and lipid rafts (DRM) and high-density fraction (HD) were separated after cell lysis with 1% TX 100 at 4 °C followed by ultracentrifugation on a discontinuous sucrose gradient. Radioactive lipids were extracted, separated with HPTLC, visualized by digital autoradiography and quantified with M3 software. Data are expressed as percentage with respect to Mock cells. Monosialosyl-diexosylganglioside (GM_3_), monosialosyl-tetraexosylganglioside (GM_1b_), disialosyl-tetraexosylganglioside (GD_1α_), gangliotetraosylceramide (GA1). **p < 0.005 vs Mock, *** *p* < 0.0005 vs Mock, °° *p* < 0.005 vs WT, °°° p < 0.0005 vs WT, ## p < 0.005 vs G1, ### p < 0.0005 vs G1

As shown in Fig. 7, the comparison among the SL pattern of ganglio-series in the DRM obtained from mock HPs or either expressing G0 or risk APOL1-Vs reveals that: i) the overexpression of the WT protein induces a reduction in the content of the GM_3_ and GD_1α_; ii) G1 causes more evident reduction of GM_3_ (around 35%), GM_1b_ is reduced by 50%, and GD_1α_ by about 40%; iii) G2 induces variations of GM_3_, which decreases by about 50%. The GA1 decreases by about 60% in podocytes overexpressing APOL1-G0 and more than 70% for G1 and G2 APOL1-Vs.

**Fig. 8:**
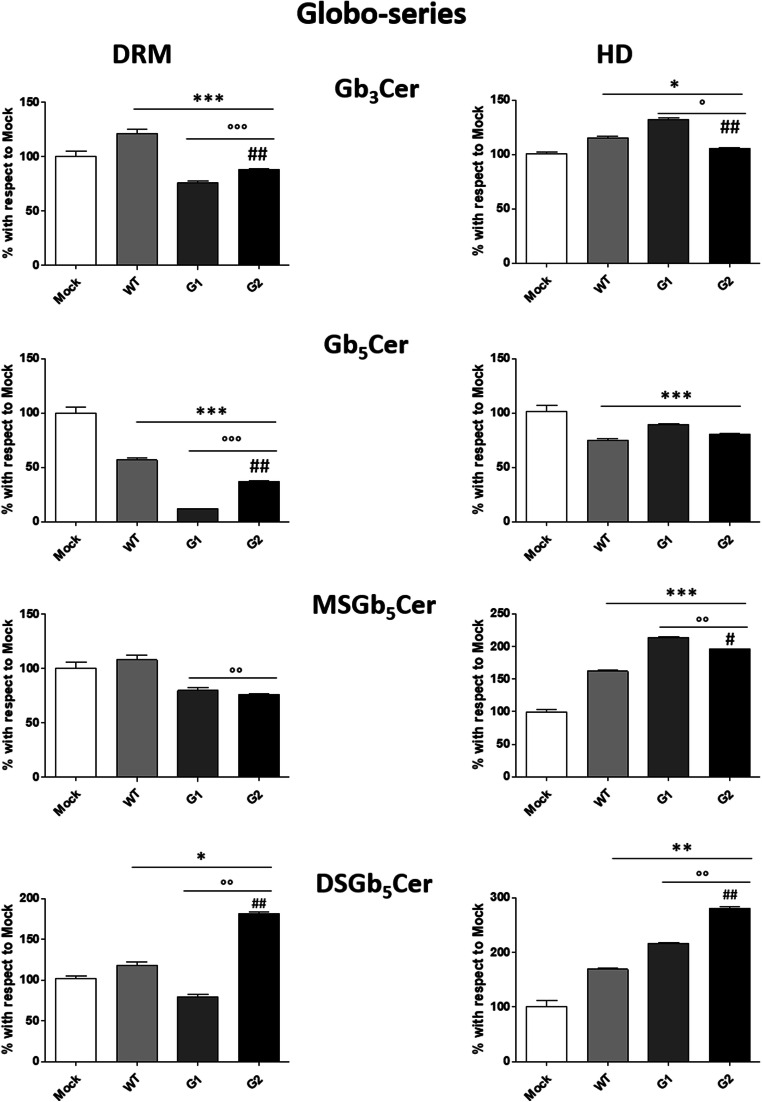
**Effects of the overexpression of APOL1 WT, G1 and G2 Vs on the globo-series content of lipid rafts.** Cell sphingolipids were metabolically labelled at the steady state with [1-^3^H]sphingosine and lipid rafts (DRM) and high-density fraction (HD) were separated, after cell lysis in 1% TX 100 at 4 °C followed by ultracentrifugation on a discontinuous sucrose gradient. Radioactive lipids were extracted, separated with HPTLC, visualized by digital autoradiography and quantified with M3 software. Data are expressed as percentage with respect to Mock cells. Globotriaosylceramide (Gb_3_Cer), globopentaosylceramide (Gb_5_Cer), monosialosyl-globopentaosylceramide (MSGb_5_Cer), disialosyl-globopentaosylceramide (DSGb_5_Cer). *p < 0.05, **p < 0.005, ***p < 0.0005, ° p < 0.005 vs WT, °° p < 0.005 vs WT, °°° p < 0.0005 vs WT, ## p < 0.005 vs G1, ## p < 0.005 vs G1

**Fig. 9:**
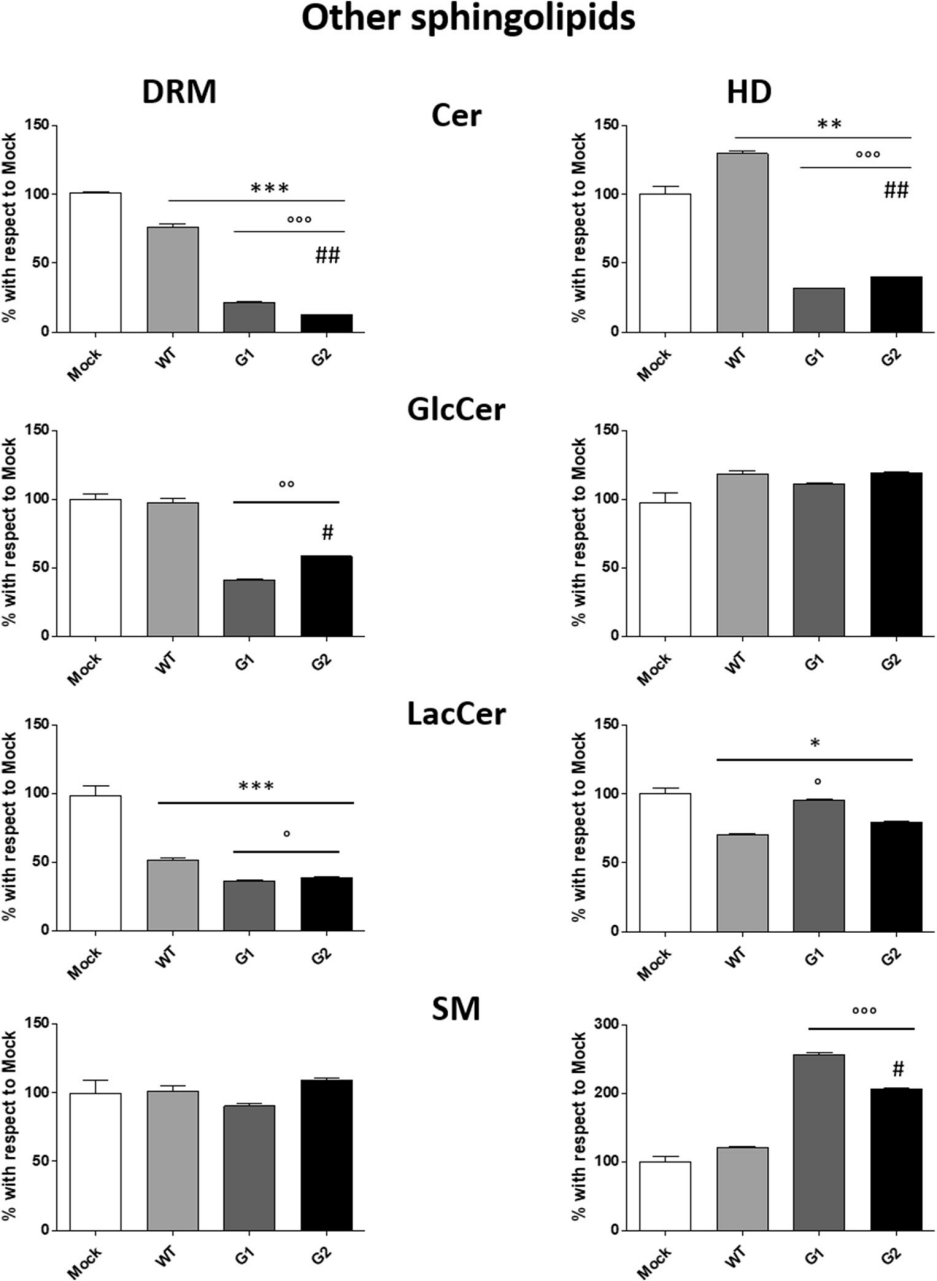
**Effects of the overexpression of APOL1 WT, G1 and G2 Vs on the other sphingolipids content of lipid rafts.** Cell sphingolipids were metabolically labelled at the steady state with [1-^3^H]sphingosine and lipid rafts (DRM) and high-α density fraction (HD) were separated after cell lysis in 1% TX 100 at 4 °C followed by ultracentrifugation on a discontinuous sucrose gradient. Radioactive lipids were extracted, separated with HPTLC, visualized by digital autoradiography and quantified with M3 software. Data are expressed as percentage with respect to Mock cells. Ceramide (Cer), glucosylceramide (GlcCer), lactosylceramide (LacCer), sphingomyelin (SM). **p < 0.005, ***p < 0.0005, ° p < 0.005 vs WT, °° p < 0.005 vs WT, °°° p < 0.0005 vs WT, # p < 0.005 vs G1, ## p < 0.005 vs G1

Significant changes due to the overexpression of all APOL1 proteins were observed in DRM also for the content of sphingolipids from globo-series (Fig. 8) and other sphingolipids (Fig. 9). Gb_3_Cer increases in WT APOL1 and decreases 25% in G1 and 18% in G2 APOL1. Gb_5_Cer decreases in WT APOL1 and G2 (44% and 60%, respectively), reaching the highest reduction in G1 APOL1 (89%). MSGb_5_Cer decreases about 20% only in the pathological APOL1-Vs. DSGb_5_Cer slightly increases in podocytes overexpressing the WT APOL1, decreases in G1 and strongly increases in G2 (81%).

As regards other SLs, the lipid rafts ceramide content decreases by about 25% on WT APOL1, a more consistent decrease was observed in G1 and G2 where it reached 80 and 88%, respectively. The levels of glucosylceramide decrease only in the DRM prepared from podocytes overexpressing the APOL1 pathological Vs. The lactosylceramide is reduced 50% in WT and 65% in G1 and G2. No changes among the mock and APOL1-Vs were observed for the SM content in the DRM.

## Discussion

APOL1 genetic Vs are potent risk factors for HIVAN [7, 8]. Importantly, a single copy APOL1 risk allele (*i.e*., when present in a heterozygous state with the G0 allele) is sufficient to increase the risk for HIVAN [42]. The specific role of APOL1 protein in the development of HIVAN is still under investigation, however, poorly controlled HIV infection is the most potent susceptibility factor for APOL1-associated nephropathy that has been identified to date [19]. Moreover, studies of APOL1 localization in human kidney biopsies with HIVAN compared to normal kidney sections observed increased expression of APOL1 protein [43]. In fact, several reports demonstrated the direct impact of APOL1 protein on HIV infection [15, 18–21]. In particular, HIV infection in HPs is able to increase expression of APOL1 creating a positive-feedback loop enhancing infection and inflammatory response particularly relevant for APOL1 risk Vs [21]. On the other hand, several lines of evidence derived from clinical and experimental studies have provided insights into the roles of lipids and lipid-modulating proteins as key determinants of podocyte function in health and kidney disease [23].

Thus, to study the impact of APOL1 on SLs pattern in the context of HIVAN we used the established HPs model with the increased expression level of APOL1 G0/WT or either G1 or G2 risk Vs.

Of note, podocytes are characterized by a peculiar lipid pattern especially for the SLs composition. Indeed, besides to classical SLs present in somatic cells such as GM_3_ and SM, HPs express several unusual SLs including GM_1b_, GD_1α_, and globopentaosylceramide (Gb_5_Cer) with its sialylated monosialosyl-globopentaosylceramide (MSGb_5_Cer) and disialosyl-globopentaosylceramide (DSGb_5_Cer). SLs due to their physical and chemical properties segregates at the plasma membrane level forming the so-called DRM. Despite SLs, DRM are enriched in cholesterol and in a specialized class of proteins able to form different macromolecular complexes that not only have a structural role, but also are an active player in the control of cell signalling [44]. The importance of DRM in the glomerular slit-diaphragm was recognized several years ago when proteins express in podocytes such as nephrin and podocin were found to be enriched in DRM [23]. Moreover, in podocytes lipid rafts-associated ganglioside GM_3_ is a receptor for the soluble Flt1 protein which binding is essential for autocrine preservation of the podocyte actin cytoskeleton and prevention of proteinuria [26]. On the other hand, alteration in the SLs composition, which in turn induces impairment of the lipid’s rafts structure, was reported in several kidney-related pathologies. In the podocytes of patients with Fabry disease, the accumulation of globotriaosylceramide (Gb_3_Cer) is associated with proteinuria and podocyte injury [45]. In Sandhoff disease, the accumulation of the specific ganglioside GM_2_ results in the alteration of the podocytes function [46].

Several intracellular and extracellular metabolic pathways and proteins contribute to the modification of SL pattern in DRM. By the comparison of the expression of the main sialytransferases involved in the SL biosynthesis and of the sialidases Neu1, Neu2, Neu3 and Neu4 we did not find statistically significative differences among these cell lines. These data together with those obtained by the analysis of the glycohydrolases in total cell lysate and at the plasma membrane level suggest that APOL1 probably interfere with the cellular trafficking of the SL and of their metabolic enzymes rather than acting on the general metabolic machinery [23]. A specific role of the intracellularly expressed APOL1 protein in HP is still under investigation, however, it is enriched in the plasma membrane and in Rab5 expressing endosomal vesicles [21]. Additionally, expression of APOL1 in HP increases upon cytokines’ stimulation such as IFN-γ and TNF-α, thus indicating its role in the in anti-viral response [47]. Overexpression of all APOL1 proteins in HPs caused lysosomal swelling and cell death, with a more marked effect and a lower concentration threshold for the G1 and G2 risk Vs, compared to the non-risk WT-APOL1 [18]. The present study sought to examine the *APOL1* polymorphism-dependent modification of SLs in HPs. We have found that the overexpression of APOL1 protein in HPs induces several changes in the SLs composition. In particular, the overexpressing of APOL1 risk Vs are characterized by an enrichment in lipid rafts of Gb_3_Cer and DSGb_5_Cer, while Cer, LacCer, GM_3_, GD_1α_, Gb_5_Cer, and GA1 decrease.

Importantly, we found that the expression of G1, compared to WT APOL1, induces a general reduction of all species of SLs, with the exception of SM. Differently, in the case of the expression of the specific G2 APOL1, we found a decreased content of Cer, GlcCer, LacCer, GM_3_, GA1, Gb_3_Cer, and Gb_5_Cer; whereas, GD_1α_ and DSGb_5_Cer are increased when compared to WT. These evidence let to speculate on a close correlation between APOL1-Vs G1/G2 and HIV internalization in HPs that could be responsible for an increased risk of developing HIVAN [7, 8, 30].

## Conclusions

Taken together, our data suggest that APOL1 is an important player in the definition of the plasma membrane SL composition, in particular within lipids rafts. In addition, since lipids rafts are direct PM player involved in HIV internalization, the alteration of their SL profile due to the expression of APOL1 G1 or G2 Vs could be responsible for the onset of HIVAN. However, further studies are necessary to dissect the specific mechanism of action of APOL1 as SL modulators as well as to correlate the alteration in podocytes SL pattern with the onset of APOL1-related pathologies.

## Methods

### Cell culture

Conditionally immortalized human podocytes (HPs) were developed and cultured as described in Saleem M.A. *et al*. [34]. Stable G0/G1/G2 APOL1 transfected HPs were generated by retroviral infection as described [21]. Briefly, the open reading frame APOL1 (G0, G1, G2) was cloned into the retroviral vector pBABE carrying resistance to puromycin. To generate retroviral particles, the viral packaging cell line HEK-GP were co-transfected with the pBABE construct of interest and the VSV gene. HPs were infected twice within 24 h with the viral-containing supernatant of HEK-GP cells. Selection with puromycin (1 μg/mL) was continued for a week, and comparable expression of respective sequence of the corresponding APOL1 was verified.

### Determination of protein content by DC protein assay

Samples protein content was determined through DC protein assay (Bio-Rad) following the manufacturer indications.

### SDS-PAGE and Western blotting

Aliquots of cell lysates corresponding to the same amount of proteins were resuspended in Laemmli buffer and denaturated for 10 min at 100 °C. SDS-PAGE was performed using a Miniprotean II unit, produced by Bio-Rad using a gradient gel of 4–20% of poly-acrylamide (Bio-Rad).

After electrophoresis separation, proteins were transferred into PVDF membrane that were in 5% skim milk in TBS-T 0,01%. Then the PVDF was washed three times with TBS-T 0.01% and incubated overnight at 4 °C with the appropriate primary antibodies. Caveolin-1(BD Transduction Laboratories) and Calnexin (Cell Signalling) antibody were used at the final dilution of 1:1000. At the end, the PVDFs were washed three times with TBS-T 0,1% and incubated for 1 h at RT with the appropriate secondary antibodies. The membranes were then washed again for three times and the peroxidase activity was assessed through incubation with horseradish peroxidase substrate (Westar Cyanagen). The chemiluminescent signal was revealed using a Mini HD9 (UviTec, Cambridge) and analyzed by Nine Alliance mini HD9 software.

### Treatment of cells with [1–^3^H]sphingosine

[1-^3^H]sphingosine dissolved in methanol is transferred into a sterile glass tube and dried under a nitrogen stream and the residue then dissolved in an appropriate volume of pre-warmed (37 °C) cell culture medium to obtain a final concentration of (3 × 10)^−8^ M. Cells are incubated for a 2 h pulse and, after that, the medium was removed and replaced with fresh medium without radioactive sphingosine for the 48 h. At the end of the chase periods, cells were collected for the SLs analyses and DRM preparation [48, 49].

### DRM preparation

Cells were lysed in lysis buffer (1% Triton X-100, 10 mM Tris buffer, pH 7.5, 150 mM NaCl, 5 mM EDTA, 1 mM Na_3_VO_4,_ 1 mM phenylmethylsulfonyl fluoride, and 75 milliunits/ml aprotinin, 3–4 mg of cell protein/ml) and Dounce homogenized (10 strokes, tight). Cell lysate is centrifuged (5 min, 1300×g) to remove nuclei and cellular debris. The postnuclear fraction is mixed with an equal volume of 85% sucrose (*w*/*v*) in 10 mM Tris buffer (pH 7.5), placed at the bottom of a discontinuous sucrose concentration gradient (30–5%) in the same buffer, and centrifuged (17 h, 200,000×g) at 4 °C. After ultracentrifugation, eleven fractions are collected starting from the top of the tube [50, 51].

### Analysis of radioactive sphingolipids

Radioactive lipids associated with total cell lysates, DRM and high density (HD) fractions were extracted twice with chloroform/methanol 2:1 (v:v) and chloroform/methanol/water 20:10:1 (v:v); the total lipid extracts were subjected to a two-phase partitioning by adding 20% water to the lipid extract accordingly to [52, 53]. The organic phases were then submitted to alkaline treatment followed by partitioning to remove glycerophospholipids. The radioactivity associated with aqueous and organic phases was evaluated by liquid scintillation. [^3^H]sphingolipids were analyzed by HPTLC (high performance thin layer chromatography) using glass plates silica gel 60 (Merck) as stationary phase and the solvent systems used as mobile phase were: first run in chloroform-methanol 2:1, followed by a second run with the solvent systems chloroform-methanol-0.2% aqueous CaCl_2_, 50:42:11 (v:v:v), for the polar sphingolipids; a run with the solvent systems chloroform-methanol-water 110:40:6 (v:v:v) for the neutral lipids. Polar sphingolipids are also separated bi-dimensional thin layer chromatography using: a first run with the solvent systems chloroform-methanol-0.2% aqueous CaCl_2_, 50:42:11 (v:v) followed by a second orthogonal run in chloroform-methanol-0.2% aqueous CaCl_2_, 50:42:11 (v:v). Radioactive lipids were visualized by digital autoradiography (^T^Racer BetaImager; BioSpace Laboratory, Nesles la Vallée, France) and quantified using BioSpace Lab’s M3 Vision software. Identification of lipids after separation was assessed by comigration with radioactive lipid standards.

### Analyses of endogenous sphingolipids

For endogenous sphingolipid pattern, total lipid extract is subjected to a two-phase partitioning as previously described [54] resulting in the separation of an aqueous phase containing polar sphingolipids and in an organic phase containing all neutral lipids. Phospholipids present in the organic phase are then removed by a mild alkaline treatment; both aqueous phase and alkali-treated organic phase are used for HPTLC as described before. Representative images were reported in supplementary Fig. 4.

In addition, part of the samples was used for mass analyses.MS analyses are carried out using a Thermo Quest Finnigan LCQDeca ion trap mass spectrometer (FINNIGAN MAT, San Jose, CA) equipped with an ESI ion source and an Xcalibur data system and a TSP P4000 quaternary pump HPLC. Separations of all SLs (sphingolipids) are obtained on a 5 μm, 250x4mm LiChrospher 100 RP8 column (Merck).

Elution of Gb_5_Cer and its sialylated forms, and gangliosides molecular species is carried out, at a flow rate of 0.5 ml/min, using a gradient formed by the solvent system A, consisting of CH_3_CN/5 mM ammonium acetate buffer, pH 7 (15:85 by volume) and solvent B containing CH_3_CN/H_2_O (85:15 by volume). The gradient is linear from 30:70 to 20:80, by volume, of A:B, over 25 min, followed by a 5 min gradient from 20:80 to 0:100, by volume, of A:B, followed by 15 min of isocratic condition at 100% of B.

Elution of SM, Cer and other neutral SLs molecular species is carried out, at a flow rate of 0.5 ml/min, with a gradient formed by the solvent system A, consisting methanol/water (90:10 by vol), and solvent system B, consisting of methanol, both containing 5 mM ammonium acetate. The gradient elution program is as follows: 5 min with solvent A; 5 min with a linear gradient from 100% solvent A to 100% solvent B; 15 min with 100% solvent B; 5 min with a linear gradient from 100% solvent B to 100% methanol. Methanol is also used to wash the column for 10 min, followed by equilibration procedure with solvent A for 15 min.

Optimum conditions for Gb_5_Cer and its sialylated forms, and ganglioside molecular species MS analyses include sheath gas flow of 70 arbitrary units, auxiliary gas flow of 10 arbitrary units, spray voltage of 4 kV, capillary voltage of −42 V, capillary temperature of 260 °C, fragmentation voltage (used for collision induced dissociation) of 40–80%. Mass spectra are acquired over a range m/z 200–2000.

Optimum conditions for SM, Cer and other neutral SLs molecular species MS analyses include sheath gas flow of 50 arbitrary units, spray voltage of 4 kV, capillary voltage of −47 V, capillary temperature of 260 °C, fragmentation voltage (used for collision induced dissociation) of 40–60%. Mass spectra are acquired over a m/z range 200–1600.

For all experiments, source ion optics are adjusted to accomplish desolvation of ions while minimizing fragmentation.

### Sialidase treatment

For sialidase treatment, aliquots of polar sphingolipids, in a total volume of 0,1 ml of aqueous solution, were incubated for 20 h at 37 °C with 0,02 unit of *Vibrio cholera* sialidase; then the samples were dried and used for HPTLC.

### Evaluation of enzymatic activities in cell lysates

The enzymatic activities associated with total cell lysates were determined using a previously described method based on 4-Methylumbelliferone (MUB)-derived fluorogenic substrates [49, 55]: 4-methylumbelliferyl β-d-glucopyranoside (MUB-β-Glc) for β-glucocerebrosidase (GCase and NLGase), 4-methylumbelliferyl β-d-galactopyranoside (MUB-β-Gal) for β-galactosidase, 4-methylumbelliferyl α-d-galactopyranoside (MUB-α-Gal) for α-galactosidase and 4-methylumbelliferyl N-acetyl-β-d-glucuronide (MUG) for β-hexosaminidase (all from Glycosynth, Warrington, United Kingdom). In order to distinguish between GCase and NLGase activity, cell lysates were preincubated for 30 min at room temperature in McIlvaine buffer (pH 6) with 5 nM AMP-DNM (adamantane-pentyl-dNM; N-(5-adamantane-1-yl-methoxy-pentyl) deoxynojirimycin), a specific inhibitor of NLGase, or 1 mM CBE (conduritol-B-epoxide) (Sigma), a specific inhibitor of CGase. At the end of preincubation, the reaction was started with the addition of 25 μl of MUB-β-Glc at the final concentration of 6 mM. To measure β-galactosidase, α-galactosidase, and β-hexosaminidase aliquots of cell lysates were incubated with 25 μl of McIlvaine buffer (pH 5.2) containing the specific florigenic substrates at the final concentration of 500 μM. Then water was added to reach the final volume of 100 μl. At different time points, the reaction mixtures were blocked by adding 19 volumes of 0.25 M glycine (pH 10.7; MilliporeSigma). Standard free MUB were used to quantify the substrate hydrolysis. Enzymatic activity is expressed as picomoles of product per milligram of cell proteins per hour.

### Evaluation of enzymatic activity on the surface of living cells

Cells were plated in 96-well microplates at a density of 20,000 cells/well, and plasm membrane-associated activities of GCase, NLGase, α-galactosidase, β-galactosidase, and β-hexosaminidase were assessed in these cells by a high throughput cell lived-based assay as previously described [49, 55]. To distinguish between GCase and NLGase activities, cells were preincubated for 30 min at room temperature in DMEM-F12 without phenol red (Thermo Fisher Scientific) containing 5 nM AMP-DNM or 1 mM CBE, respectively [56]. Activities were assayed using the artificial MUB-α-Gal for α-galactosidase, MUB-β-Gal for β-galactosidase, MUG for β-hexosaminidase, and MUB-β-Glc for GCase and NLGase. The fluorogenic substrates were solubilized in DMEM-F12 without phenol red at pH 6, with final concentrations of 250 μM, 250 μM, 1 mM, and 6 mM, respectively. Aliquots of medium (10 μl) were analysed at different time points by a Victor microplate reader (PerkinElmer), after the addition of 190 μl of 0.25 M glycine (pH 10.7). Standard free MUB was used to construct calibration curves and quantify substrate hydrolysis. Enzymatic activity is expressed as picomoles of product per 10^6^ cells per hour.

### Whole-Transcriptome data analysis

Raw sequencing single-end reads were processed for quality check using FASTQC (v0.11.5) (*http://www.bioinformatics.babraham.ac.uk/projects/fastqc/*). Raw reads were mapped to the human reference genome (Gencode GRCh38 v24 primary assembly) using STAR aligner (v2.5.2a) [57]. Gencode annotation v24 gene transfer file (GTF) was used as reference gene annotation file for alignment. Aligned Bam data were sorted and indexed using SAMtools [58]. Gene expression was quantified by using the quantmode GeneCounts option of the STAR aligner; the counts produced using this approach coincide with those produced by HTSeq-count [59] using default parameters. The statistical analysis was performed by using DESeq2 package [60], testing (Wald Test) group vs group accordingly to experimental design. A threshold of 0.05 was applied to False Discovery Rate (FDR) adjusted *p* values in order to select the differentially expressed genes (DEGs) to use in downstream analysis. Exploration data analysis (clustering and principal component analysis - PCA) was performed using built-in functions of the DESeq2 package.

### Statistical analysis

All the experiments were performed in triplicate and repeated ≥3 times. Data are presented as the means ± S.D. and were tested for significance with Student’s *t* test or one-way ANOVA using GraphPad Prism 8 software (GraphPad Software, La Jolla, CA, USA).

## Electronic supplementary material

ESM 1 (PDF 643 kb)

## Data Availability

we declare that all the row data are available.

## References

[1] Duchateau PN, Pullinger CR, Orellana RE, Kunitake ST, Naya-Vigne J, O'Connor PM, Malloy MJ, Kane JP (1997). Apolipoprotein L, a new human high density lipoprotein apolipoprotein expressed by the pancreas. Identification, cloning, characterization, and plasma distribution of apolipoprotein L. J Biol Chem.

[2] Duchateau PN, Movsesyan I, Yamashita S, Sakai N, Hirano K, Schoenhaus SA, O'Connor-Kearns PM, Spencer SJ, Jaffe RB, Redberg RF, Ishida BY, Matsuzawa Y, Kane JP, Malloy MJ (2000). Plasma apolipoprotein L concentrations correlate with plasma triglycerides and cholesterol levels in normolipidemic, hyperlipidemic, and diabetic subjects. J. Lipid Res..

[3] Vanhamme L, Paturiaux-Hanocq F, Poelvoorde P, Nolan DP, Lins L, Van Den Abbeele J, Pays A, Tebabi P, Van Xong H, Jacquet A, Moguilevsky N, Dieu M, Kane JP, De Baetselier P, Brasseur R, Pays E (2003). Apolipoprotein L-I is the trypanosome lytic factor of human serum. Nature.

[4] Molina-Portela MP, Samanovic M, Raper J (2008). Distinct roles of apolipoprotein components within the trypanosome lytic factor complex revealed in a novel transgenic mouse model. J. Exp. Med..

[5] Perez-Morga D, Vanhollebeke B, Paturiaux-Hanocq F, Nolan DP, Lins L, Homble F, Vanhamme L, Tebabi P, Pays A, Poelvoorde P, Jacquet A, Brasseur R, Pays E (2005). Apolipoprotein L-I promotes trypanosome lysis by forming pores in lysosomal membranes. Science.

[6] Thomson R, Genovese G, Canon C, Kovacsics D, Higgins MK, Carrington M, Winkler CA, Kopp J, Rotimi C, Adeyemo A, Doumatey A, Ayodo G, Alper SL, Pollak MR, Friedman DJ, Raper J (2014). Evolution of the primate trypanolytic factor APOL1. Proc. Natl. Acad. Sci. U. S. A..

[7] Medapalli RK, He JC, Klotman PE (2011). HIV-associated nephropathy: pathogenesis. Curr. Opin. Nephrol. Hypertens..

[8] Kopp JB, Heymann J, Winkler CA (2017). APOL1 renal risk variants: fertile soil for HIV-associated nephropathy. Semin. Nephrol..

[9] Mikulak J, Singhal PC (2010). HIV-1 and kidney cells: better understanding of viral interaction. Nephron Exp Nephrol.

[10] Rednor SJ, Ross MJ (2018). Molecular mechanisms of injury in HIV-associated nephropathy. Front Med (Lausanne).

[11] Mikulak J, Teichberg S, Arora S, Kumar D, Yadav A, Salhan D, Pullagura S, Mathieson PW, Saleem MA, Singhal PC (2010). DC-specific ICAM-3-grabbing nonintegrin mediates internalization of HIV-1 into human podocytes. Am J Physiol Renal Physiol.

[12] Wan G, Zhaorigetu S, Liu Z, Kaini R, Jiang Z, Hu CA (2008). Apolipoprotein L1, a novel Bcl-2 homology domain 3-only lipid-binding protein, induces autophagic cell death. J. Biol. Chem..

[13] Zhaorigetu S, Wan G, Kaini R, Jiang Z, Hu CA (2008). ApoL1, a BH3-only lipid-binding protein, induces autophagic cell death. Autophagy.

[14] Ma L, Shelness GS, Snipes JA, Murea M, Antinozzi PA, Cheng D, Saleem MA, Satchell SC, Banas B, Mathieson PW, Kretzler M, Hemal AK, Rudel LL, Petrovic S, Weckerle A, Pollak MR, Ross MD, Parks JS, Freedman BI (2015). Localization of APOL1 protein and mRNA in the human kidney: nondiseased tissue, primary cells, and immortalized cell lines. J. Am. Soc. Nephrol..

[15] Taylor HE, Khatua AK, Popik W (2014). The innate immune factor apolipoprotein L1 restricts HIV-1 infection. J. Virol..

[16] Kumar V, Ayasolla K, Jha A, Mishra A, Vashistha H, Lan X, Qayyum M, Chinnapaka S, Purohit R, Mikulak J, Saleem MA, Malhotra A, Skorecki K, Singhal PC (2019). Disrupted apolipoprotein L1-miR193a axis dedifferentiates podocytes through autophagy blockade in an APOL1 risk milieu. Am J Physiol Cell Physiol.

[17] Kumar V, Vashistha H, Lan X, Chandel N, Ayasolla K, Shoshtari SSM, Aslam R, Paliwal N, Abbruscato F, Mikulak J, Popik W, Atta MG, Chander PN, Malhotra A, Meyer-Schwesinger C, Skorecki K, Singhal PC (2018). Role of Apolipoprotein L1 in human parietal epithelial cell transition. Am. J. Pathol..

[18] Lan X, Jhaveri A, Cheng K, Wen H, Saleem MA, Mathieson PW, Mikulak J, Aviram S, Malhotra A, Skorecki K, Singhal PC (2014). APOL1 risk variants enhance podocyte necrosis through compromising lysosomal membrane permeability. Am J Physiol Renal Physiol.

[19] Lan X, Wen H, Lederman R, Malhotra A, Mikulak J, Popik W, Skorecki K, Singhal PC (2015). Protein domains of APOL1 and its risk variants. Exp. Mol. Pathol..

[20] Lan X, Wen H, Saleem MA, Mikulak J, Malhotra A, Skorecki K, Singhal PC (2015). Vascular smooth muscle cells contribute to APOL1-induced podocyte injury in HIV milieu. Exp. Mol. Pathol..

[21] Mikulak J, Oriolo F, Portale F, Tentorio P, Lan X, Saleem MA, Skorecki K, Singhal PC, Mavilio D (2016). Impact of APOL1 polymorphism and IL-1beta priming in the entry and persistence of HIV-1 in human podocytes. Retrovirology.

[22] Hakomori Si SI (2002). The glycosynapse. Proc. Natl. Acad. Sci. U. S. A..

[23] Fornoni A, Merscher S, Kopp JB (2014). Lipid biology of the podocyte--new perspectives offer new opportunities. Nat Rev Nephrol.

[24] Wei C, Moller CC, Altintas MM, Li J, Schwarz K, Zacchigna S, Xie L, Henger A, Schmid H, Rastaldi MP, Cowan P, Kretzler M, Parrilla R, Bendayan M, Gupta V, Nikolic B, Kalluri R, Carmeliet P, Mundel P, Reiser J (2008). Modification of kidney barrier function by the urokinase receptor. Nat. Med..

[25] Yamashita T, Hashiramoto A, Haluzik M, Mizukami H, Beck S, Norton A, Kono M, Tsuji S, Daniotti JL, Werth N, Sandhoff R, Sandhoff K, Proia RL (2003). Enhanced insulin sensitivity in mice lacking ganglioside GM3. Proc. Natl. Acad. Sci. U. S. A..

[26] Jin J, Sison K, Li C, Tian R, Wnuk M, Sung HK, Jeansson M, Zhang C, Tucholska M, Jones N, Kerjaschki D, Shibuya M, Fantus IG, Nagy A, Gerber HP, Ferrara N, Pawson T, Quaggin SE (2012). Soluble FLT1 binds lipid microdomains in podocytes to control cell morphology and glomerular barrier function. Cell.

[27] Galeano B, Klootwijk R, Manoli I, Sun M, Ciccone C, Darvish D, Starost MF, Zerfas PM, Hoffmann VJ, Hoogstraten-Miller S, Krasnewich DM, Gahl WA, Huizing M (2007). Mutation in the key enzyme of sialic acid biosynthesis causes severe glomerular proteinuria and is rescued by N-acetylmannosamine. J. Clin. Invest..

[28] Merscher S, Fornoni A (2014). Podocyte pathology and nephropathy - sphingolipids in glomerular diseases. Front Endocrinol (Lausanne).

[29] Charest PM, Roth J (1985). Localization of sialic acid in kidney glomeruli: regionalization in the podocyte plasma membrane and loss in experimental nephrosis. Proc. Natl. Acad. Sci. U. S. A..

[30] Mikulak J, Singhal PC (2010). HIV-1 entry into human podocytes is mediated through lipid rafts. Kidney Int.

[31] Varchetta S, Lusso P, Hudspeth K, Mikulak J, Mele D, Paolucci S, Cimbro R, Malnati M, Riva A, Maserati R, Mondelli MU, Mavilio D (2013). Sialic acid-binding Ig-like lectin-7 interacts with HIV-1 gp120 and facilitates infection of CD4pos T cells and macrophages. Retrovirology.

[32] Abou Daher, A., El Jalkh, T., Eid, A.A., Fornoni, A., Marples, B., Zeidan, Y.H.: Translational Aspects of Sphingolipid Metabolism in Renal Disorders. Int J Mol Sci. **18**(12), (2017). 10.3390/ijms18122528 10.3390/ijms18122528 PMC5751131 29186855

[33] Bruggeman LA, Dikman S, Meng C, Quaggin SE, Coffman TM, Klotman PE (1997). Nephropathy in human immunodeficiency virus-1 transgenic mice is due to renal transgene expression. J. Clin. Invest..

[34] Saleem MA, O'Hare MJ, Reiser J, Coward RJ, Inward CD, Farren T, Xing CY, Ni L, Mathieson PW, Mundel P (2002). A conditionally immortalized human podocyte cell line demonstrating nephrin and podocin expression. J. Am. Soc. Nephrol..

[35] Kopp JB, Klotman ME, Adler SH, Bruggeman LA, Dickie P, Marinos NJ, Eckhaus M, Bryant JL, Notkins AL, Klotman PE (1992). Progressive glomerulosclerosis and enhanced renal accumulation of basement membrane components in mice transgenic for human immunodeficiency virus type 1 genes. Proc. Natl. Acad. Sci. U. S. A..

[36] Hirabayashi Y, Hyogo A, Nakao T, Tsuchiya K, Suzuki Y, Matsumoto M, Kon K, Ando S (1990). Isolation and characterization of extremely minor gangliosides, GM1b and GD1 alpha, in adult bovine brains as developmentally regulated antigens. J. Biol. Chem..

[37] Kannagi R, Cochran NA, Ishigami F, Hakomori S, Andrews PW, Knowles BB, Solter D (1983). Stage-specific embryonic antigens (SSEA-3 and -4) are epitopes of a unique globo-series ganglioside isolated from human teratocarcinoma cells. EMBO J..

[38] Saito S, Levery SB, Salyan ME, Goldberg RI, Hakomori S (1994). Common tetrasaccharide epitope NeuAc alpha 2-->3Gal beta 1-->3(Neu-ac alpha 2-->6)GalNAc, presented by different carrier glycosylceramides or O-linked peptides, is recognized by different antibodies and ligands having distinct specificities. J. Biol. Chem..

[39] Saito S, Aoki H, Ito A, Ueno S, Wada T, Mitsuzuka K, Satoh M, Arai Y, Miyagi T (2003). Human alpha2,3-sialyltransferase (ST3Gal II) is a stage-specific embryonic antigen-4 synthase. J. Biol. Chem..

[40] Reivinen J, Holthofer H, Miettinen A (1992). A cell-type specific ganglioside of glomerular podocytes in rat kidney: an O-acetylated GD3. Kidney Int..

[41] Holthofer H, Reivinen J, Miettinen A (1994). Nephron segment and cell-type specific expression of gangliosides in the developing and adult kidney. Kidney Int..

[42] Kasembeli AN, Duarte R, Ramsay M, Mosiane P, Dickens C, Dix-Peek T, Limou S, Sezgin E, Nelson GW, Fogo AB, Goetsch S, Kopp JB, Winkler CA, Naicker S (2015). APOL1 risk variants are strongly associated with HIV-associated nephropathy in black south Africans. J. Am. Soc. Nephrol..

[43] Madhavan SM, O'Toole JF, Konieczkowski M, Ganesan S, Bruggeman LA, Sedor JR (2011). APOL1 localization in normal kidney and nondiabetic kidney disease. J. Am. Soc. Nephrol..

[44] Sezgin E, Levental I, Mayor S, Eggeling C (2017). The mystery of membrane organization: composition, regulation and roles of lipid rafts. Nat Rev Mol Cell Biol.

[45] Najafian B, Svarstad E, Bostad L, Gubler MC, Tondel C, Whitley C, Mauer M (2011). Progressive podocyte injury and globotriaosylceramide (GL-3) accumulation in young patients with Fabry disease. Kidney Int..

[46] Tatematsu M, Imaida K, Ito N, Togari H, Suzuki Y, Ogiu T (1981). Sandhoff disease. Acta Pathol. Jpn..

[47] Nichols B, Jog P, Lee JH, Blackler D, Wilmot M, D'Agati V, Markowitz G, Kopp JB, Alper SL, Pollak MR, Friedman DJ (2015). Innate immunity pathways regulate the nephropathy gene Apolipoprotein L1. Kidney Int..

[48] Chigorno V, Palestini P, Sciannamblo M, Dolo V, Pavan A, Tettamanti G, Sonnino S (2000). Evidence that ganglioside enriched domains are distinct from caveolae in MDCK II and human fibroblast cells in culture. Eur. J. Biochem..

[49] Malekkou, A., Samarani, M., Drousiotou, A., Votsi, C., Sonnino, S., Pantzaris, M., Chiricozzi, E., Zamba-Papanicolaou, E., Aureli, M., Loberto, N., Christodoulou, K.: Biochemical Characterization of the GBA2 c.1780G>C Missense Mutation in Lymphoblastoid Cells from Patients with Spastic Ataxia. Int J Mol Sci. **19**(10), (2018). 10.3390/ijms19103099 10.3390/ijms19103099 PMC6213336 30308956

[50] Prinetti A, Chigorno V, Tettamanti G, Sonnino S (2000). Sphingolipid-enriched membrane domains from rat cerebellar granule cells differentiated in culture. A compositional study. J Biol Chem.

[51] Mikulak J, Di Vito C, Zaghi E, Mavilio D (2017). Host immune responses in HIV-1 infection: the emerging pathogenic role of Siglecs and their clinical correlates. Front. Immunol..

[52] Valsecchi M, Aureli M, Mauri L, Illuzzi G, Chigorno V, Prinetti A, Sonnino S (2010). Sphingolipidomics of A2780 human ovarian carcinoma cells treated with synthetic retinoids. J. Lipid Res..

[53] Samarani M, Loberto N, Solda G, Straniero L, Asselta R, Duga S, Lunghi G, Zucca FA, Mauri L, Ciampa MG, Schiumarini D, Bassi R, Giussani P, Chiricozzi E, Prinetti A, Aureli M, Sonnino S (2018). A lysosome-plasma membrane-sphingolipid axis linking lysosomal storage to cell growth arrest. FASEB J..

[54] Di Biase E, Lunghi G, Fazzari M, Maggioni M, Pome DY, Valsecchi M, Samarani M, Fato P, Ciampa MG, Prioni S, Mauri L, Sonnino S, Chiricozzi E (2020). Gangliosides in the differentiation process of primary neurons: the specific role of GM1-oligosaccharide. Glycoconj. J..

[55] Aureli M, Loberto N, Lanteri P, Chigorno V, Prinetti A, Sonnino S (2011). Cell surface sphingolipid glycohydrolases in neuronal differentiation and aging in culture. J. Neurochem..

[56] Overkleeft HS, Renkema GH, Neele J, Vianello P, Hung IO, Strijland A, van der Burg AM, Koomen GJ, Pandit UK, Aerts JM (1998). Generation of specific deoxynojirimycin-type inhibitors of the non-lysosomal glucosylceramidase. J. Biol. Chem..

[57] Dobin A, Davis CA, Schlesinger F, Drenkow J, Zaleski C, Jha S, Batut P, Chaisson M, Gingeras TR (2013). STAR: ultrafast universal RNA-seq aligner. Bioinformatics.

[58] Li H, Handsaker B, Wysoker A, Fennell T, Ruan J, Homer N, Marth G, Abecasis G, Durbin R (2009). Genome project data processing, S.: the sequence alignment/map format and SAMtools. Bioinformatics.

[59] Anders S, Pyl PT, Huber W (2015). HTSeq--a Python framework to work with high-throughput sequencing data. Bioinformatics.

[60] Love MI, Huber W, Anders S (2014). Moderated estimation of fold change and dispersion for RNA-seq data with DESeq2. Genome Biol..

