# Supplementary material for: APOL1 polymorphism modulates sphingolipid profile of human podocytes
Source: Glycoconj J. 2020 Sep 11;37(6):729–44. doi: 10.1007/s10719-020-09944-w (PMC7679335; doi:10.1007/s10719-020-09944-w)
Supplement: Supplementary file 1 — (PDF 643 kb) [file 10719_2020_9944_MOESM1_ESM.pdf]

Supplementary table 1. Sphingolipids pattern in HPs

Abbreviation	Full name IUPAC-IUB	Structure
GM_3	II^3Neu5AcLacCer	α-Neu5Ac-(2-3)-β-Gal-(1-4)-β-Glc-(1-1)-Cer
$MSGb_5$Cer	V^3Neu5AcGb_5Cer	α-Neu5Ac-(2-3)-ß-Gal-(1-3)-ß-GalNAc-(1-3)-α-Gal-(1-4)-ß-Gal-(1-4)-ß-Glc-(1-1)-Cer
GM_{1b}	IV^3Neu5AcGg_4Cer	α-Neu5Ac-(2-3)-β-Gal-(1-3)-β-GalNAc-(1-4)-β-Gal-(1-4)-β-Glc-(1-1)-Cer
$DSGb_5$Cer	V^3Neu5AcIV^6Neu5AcGb_5Cer	α-Neu5Ac-(2-3)-ß-Gal-(1-3)-[α-Neu5Ac-(2-6)-]ß-GalNAc-(1-3)-α-Gal-(1-4)-ß-Gal-(1-4)-ß-Glc-(1-1)-Cer
$GD_{1\alpha}$	IV^3Neu5AcIII^6Neu5AcGg_4Cer	α-Neu5Ac-(2-3)-β-Gal-(1-3)-[α-Neu5Ac-(2-6)-]β-GalNAc-(1-4)-β-Gal-(1-4)-β-Glc-(1-1)-Cer
Gb_5Cer	globopentaosylceramide, Gb_5Cer,	ß-Gal-(1-3)-ß-GalNAc-(1-3)-α-Gal-(1-4)-ß-Gal-(1-4)-ß-Glc-(1-1)-Cer
GA1	Gg_4Cer	β-Gal-(1-3)-β-GalNAc-(1-4)-β-Gal-(1-4)-β-Glc-(1-1)-Cer
Cer	Ceramide,Cer	Cer
GlcCer	Glucosylceramide, GlcCer	β-Glc-(1-1)-Cer
LacCer	Lactosylceramide, LacCer	β-Gal-(1-4)-β-Glc-(1-1)-Cer
Gb_3Cer	Globotriaosylceramide, Gb_3Cer	α-Gal-(1-4)-β-Gal-(1-4)-β-Glc-(1-1)-Cer
SM	Sphingomyelin	ceramide 1-phosphocholine

Supplementary figure 1.

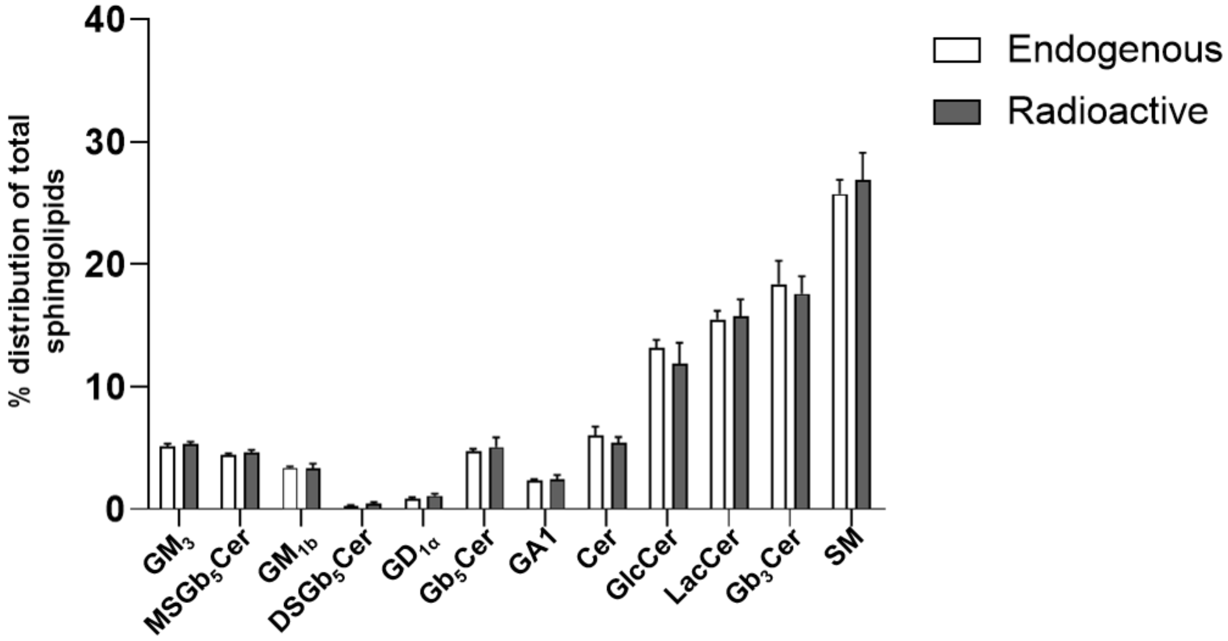

Supplementary figure 1. Sphingolipid molecular species and distribution in human podocytes. Cell sphingolipids were metabolically labelled at the steady state with [1-^{3}H]sphingosine, separated by HPTLC, visualized by digital autoradiography and quantified with M3 software. In the graphs, data were reported as percentage of the radioactive lipid distribution compared with the endogenous counterpart. Gangliotetraosylceramide (GA1), globotriaosylceramide (Gb_3Cer), globopentaosylceramide (Gb_5Cer), monosialosyl-globopentaosylceramide ($MSGb_5Cer$), disialosyl-globopentaosylceramide ($DSGb_5Cer$), ceramide (Cer), glucosylceramide (GlcCer), lactosylceramide (LacCer), sphingomyelin (SM).

Supplementary figure 2.

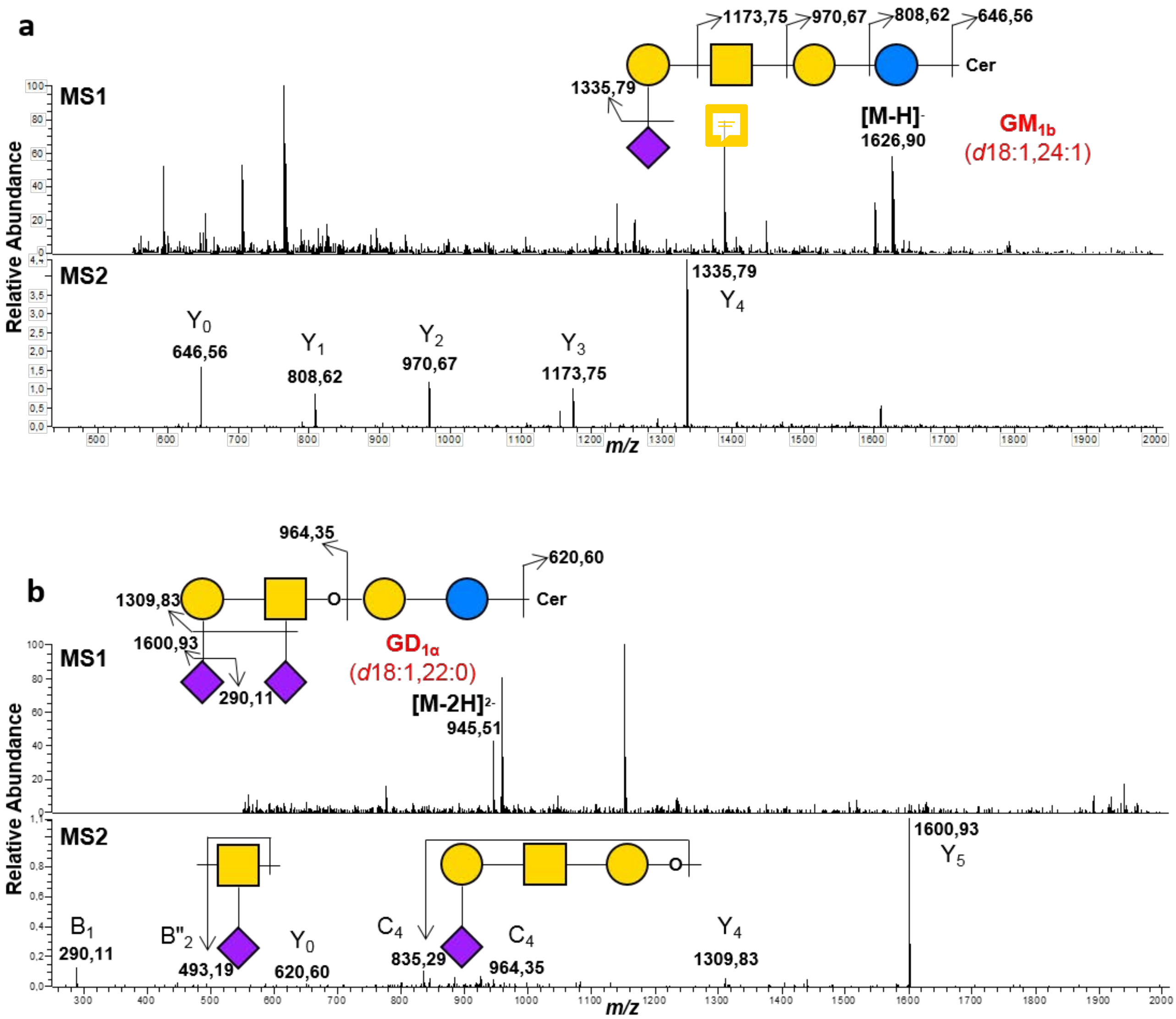

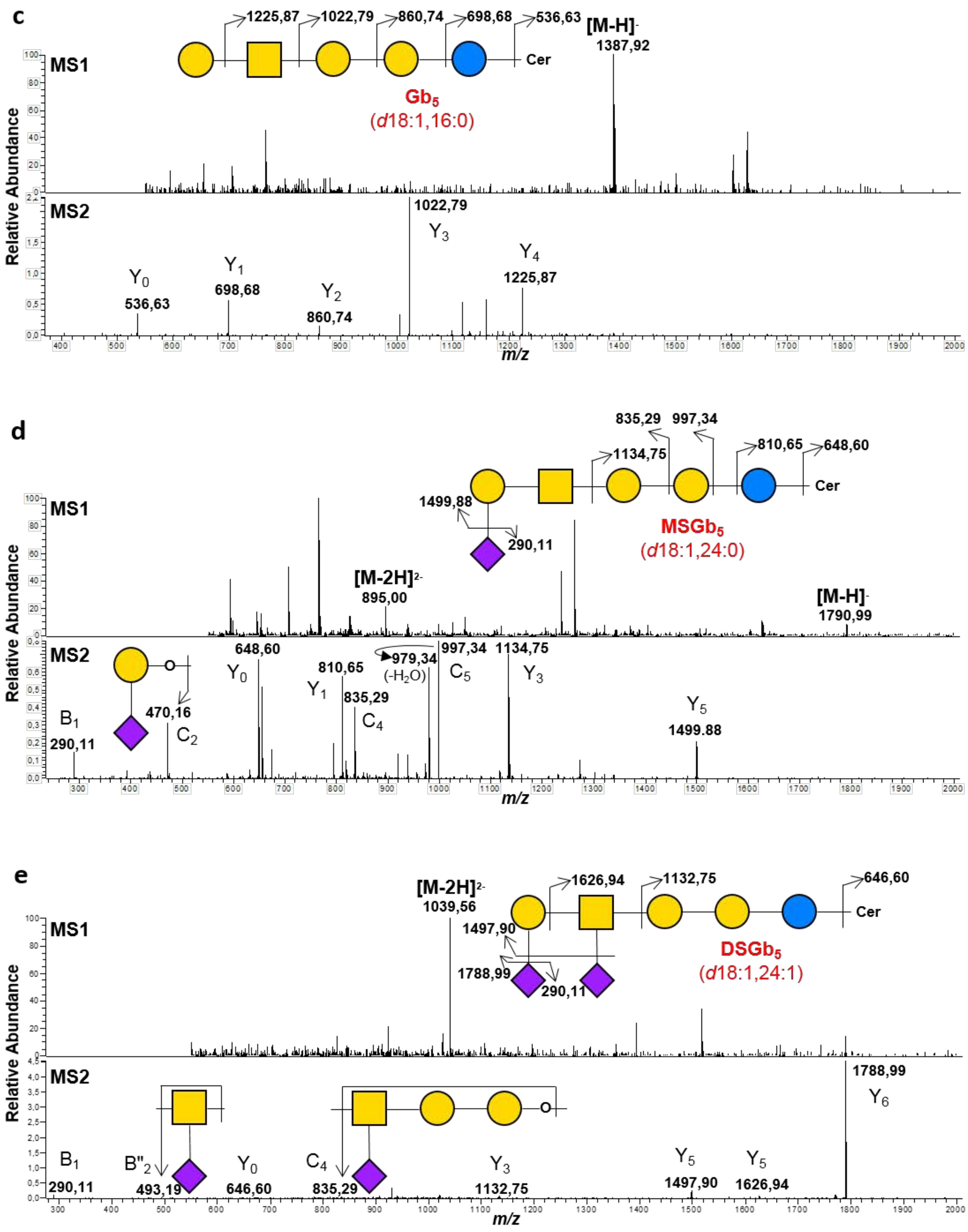

Supplementary figure 2. Mass spectrometry characterization of SLs identified in HPs. Lipids were extracted from cells, subjected to partitioning and all polar sphingolipids present in the aqueous phases were analyzed by HPLC-ESI-MS. For simplicity, only some representative spectra are shown. (a) Negative HPLC-ESI-MS1 spectrum of the GM_{1b}(d18:1,24:1) species together with the MS2 spectrum derived from the molecular ion at m/z 1626,90. (b) Negative HPLC-ESI-MS1 spectrum of the $GD_{1\alpha}$(d18:1,22:0) species together with the MS2 spectrum derived from the doubly

charged ion at m/z 945,51. (c) Negative HPLC-ESI-MS1 spectrum of the Gb_5Cer(d18:1,16:0) species together with the MS2 spectrum derived from the molecular ion at m/z 1387,92. (d) Negative HPLC-ESI-MS1 spectrum of the $MSGb_5Cer$(d18:1,24:0) species together with the MS2 spectrum derived from the doubly charged ion at m/z 895,00. (e) Negative HPLC-ESI-MS1 spectrum of the $DSGb_5Cer$(d18:1,24:1) species together with the MS2 spectrum derived from the doubly charged ion at m/z 1039,56. Assignment of major fragment ions are illustrated by the embedded drawings; some fragments have been moved below to make Figure 2 more readable

Supplementary figure 3.

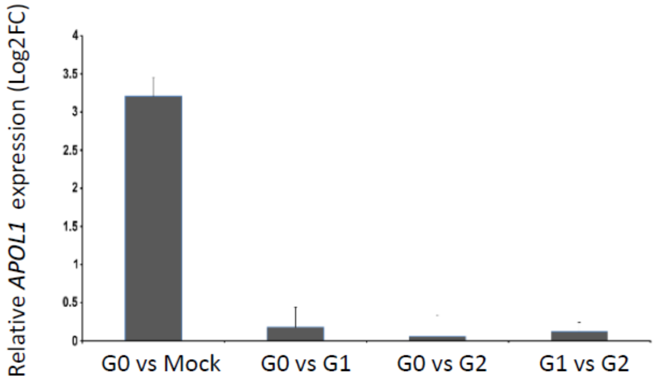

Supplementary figure 3. Relative *APOL1* gene expression level analyzed in HPs stably transfected with empty vector (Mock) or with WT APOL1 (G0), or G1 and G2 risk Vs in whole-transcriptome HPs data.

Supplementary figure 4.

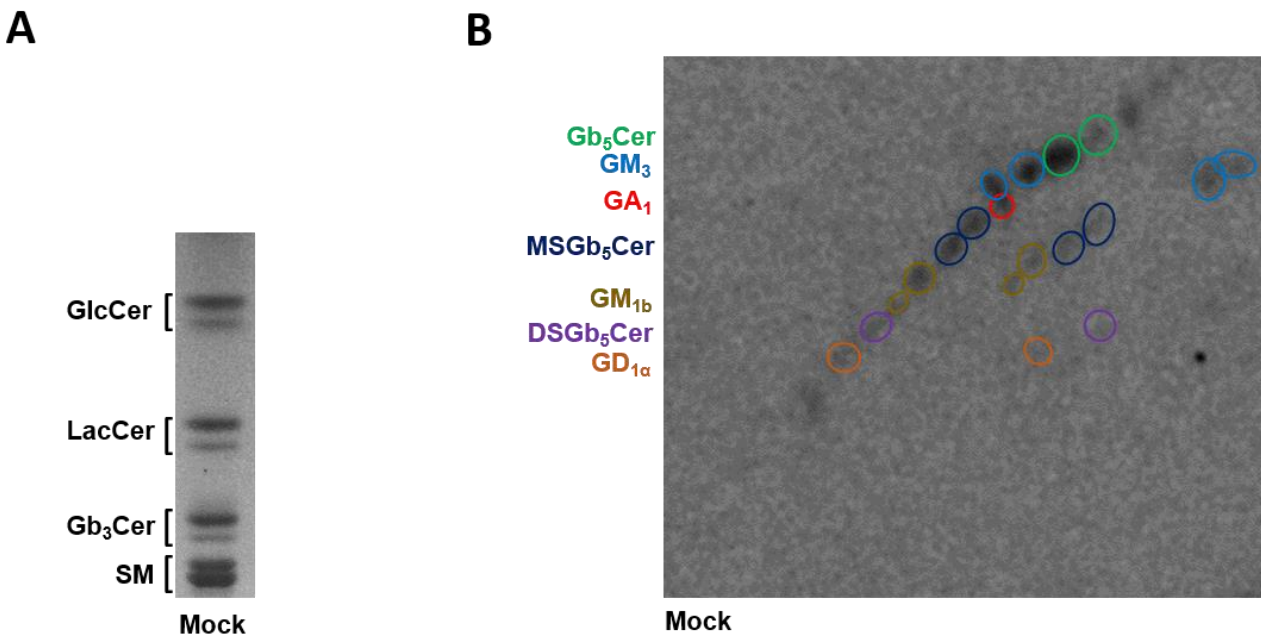

Supplementary figure 4. Representative HPTLC images of lipids contained in Mock cells.

A) lipids contained in organic phase of Mock hepatocytes separated by HPTLC using the solvent system chloroform-methanol-water 110:40:6 (v:v:v) and visualized by anisaldehyde reagent (SM: sphingomyelin, Gb_3Cer: globotriaosylceramide, LacCer: lactosylceramide, GlcCer: glucosylceramide. B) polar lipids identified by Bi-dimensional HPTLC and sialidase treatment. Bi-dimensional chromatography was performed using a first run with the solvent systems chloroform-methanol-0.2% aqueous CaCl2, 50:42:11 (v:v:v) followed by a second orthogonal run in chloroform-methanol-0.2% aqueous CaCl[illegible]0:42:11 (v:v). After the first run, 0.02 units of Vibrio cholera sialidase in 0.15ml of distilled water were stratified on the separated sphingolipids in the first run (three times during the day), and incubated for 8 hours at 37°C in a humidified incubator. Before the second run, the plate was dried for 48 hours in anhydrous environment. Lipids were visualized using anisaldehyde reagent (Gb5[illegible]: globopentaosylceramide, G[illegible]: monosialosyl-diexosylganglioside, GA1: gangliotetraosylceramide, MSGb[illegible]r: monosialosyl-globopentaosylceramide, GM_{1b}: monosialosyl-tetraexosylganglioside, DSGb[illegible]er: [illegible]isialosyl-globopentaosylceramide, GD[illegible] disialosyl-tetraexosylganglioside.

To note all the lipids outside the diagonal site in the Bi-dimensional HPTLC are desialylated due to sialidase treatment.
